# MicroRNA-181a promotes tumor growth and liver metastasis in colorectal cancer by targeting the tumor suppressor WIF-1

**DOI:** 10.1186/1476-4598-13-86

**Published:** 2014-04-23

**Authors:** Dengbo Ji, Zhiguo Chen, Ming Li, Tiancheng Zhan, Yunfeng Yao, Zhiqian Zhang, Jianzhong Xi, Li Yan, Jin Gu

**Affiliations:** 1 Key laboratory of Carcinogenesis and Translational Research (Ministry of Education), Department of Colorectal Surgery, Peking University Cancer Hospital & Institute, No. 52 Fucheng Rd., Haidian District, Beijing 100142, China; 2 Department of Cell Biology, Peking University Cancer Hospital & Institute, No. 52 Fucheng Rd., Haidian District, Beijing 100142, China; 3 Department of Biomedical Engineering, College of Engineering, Peking University, Beijing 100871, China

**Keywords:** miR-181a, Colorectal cancer, Liver metastasis, WIF-1

## Abstract

**Background:**

Given the emerging role of microRNA in tumor disease progression, we investigated the association between microRNA expression, liver metastasis and prognosis of colorectal cancer.

**Methods:**

Colorectal cancer tissues from patients with or without liver metastases were profiled to identify differentially expressed microRNA. Expression profile was further assessed using quantitative reverse transcription PCR and in situ hybridization. Correlation between miR-181a expression, the most differentially expressed microRNA, between patients with and without liver metastasis, and its downstream target genes were investigated using qRT-PCR. Luciferase reporter assay was conducted to establish functional association between miR-181a and its target genes. Manipulation of miR-181a expression and its consequences in tumor growth and metastasis were demonstrated in various *in vitro* and *in vivo* models.

**Results:**

miR-181a was revealed being the most elevated in CRC with liver metastases. miR-181a expression correlated with advanced stage, distant metastasis, and served as an independent prognostic factor of poor overall survival. Stable transfection of CRC cell lines with miR-181a promoted cell motility and invasion, as well as tumor growth and liver metastasis,while silencing its expression resulted in reduced migration and invasion. Additionally, we identified WIF-1 as direct and functional targets of miR-181a. Ectopic expression of miR-181a suppressed the epithelial markers E-cadherin and β-catenin, while enhanced the mesenchymal markers vimentin.

**Conclusion:**

Our data demonstrate that miR-181a expression is associated with CRC liver metastasis and survival. miR-181a has strong tumor-promoting effects through inhibiting the expression of WIF-1, and its potential role in promoting epithelial-mesenchymal transition.

## Introduction

Colorectal cancer (CRC) is the third most prevalent cancer worldwide [1]. Approximately 50% of CRC patients die from distant metastases, in particular liver metastases. Most CRC patients with liver metastasis are not candidates for surgical treatment, with 5-year survival rate below 10% [2]. New diagnostic means to detect early metastasis and to predict metastatic risk in CRC are in urgent demand.

microRNAs (miRNAs) are small, non-coding RNA that silence specific target genes in mammalian cells by repressing translation [3]. miRNAs play important roles in tumor invasion and metastasis [4]. Depending on its target genes, an miRNA can function either as an oncogene or a tumor suppressive gene [5]. Dysfunction of miRNA is associated with CRC tumorigenesis and progression. Furthermore, aberrant expression of specific miRNA may be used as potential prognostic and predictive markers in CRC [6-8]. However, more extensive research is required to elucidate molecular mechanisms of miRNAs that mediate CRC liver metastasis and to identify those miRNAs that may be employed as novel prognostic predictors.

In the present study, microarray-based strategy was applied to identify differentially expressed miRNA in CRC by comparing miRNA profiles between colorectal cancer tissues from patients with and without liver metastases. miR-181a was most significantly up-regulated in cancerous tissues with liver metastasis compared to without liver metastasis. Its tumor promoting function and mechanism were further characterized.

## Results

### miRNAs are differentially expressed in CRC tumor tissues from patients with or without liver metastasis

To investigate the role of miRNA in CRC liver metastasis, miRNA expression was profiled in CRC tumor tissue from patients with (n = 5) or without (n = 5) liver metastases. Among the 743 miRNA probes on Mammalian miRNA arrayV2.0, 214 miRNAs were detected in CRC tissues. hsa-miR-181a was detected to be most elevated with > 2-fold between CRC with and without liver metastasis. (Additional file 1: Table S1).

The microarray results were validated with quantitative reverse transcription PCR (qRT-PCR) by confirming the expression of hsa-miR-181a in the same 10 human CRC tissues. All further analyses in this study focused on miR-181a.

### Deregulated expression of miR-181a is associated with liver metastasis in CRC patients

To verify the microarray results of differentiated miRNA expression, we performed qRT-PCR analyses in a cohort of 137 paired CRC and normal tissues (Validation Cohort 1).

**Figure 1:**
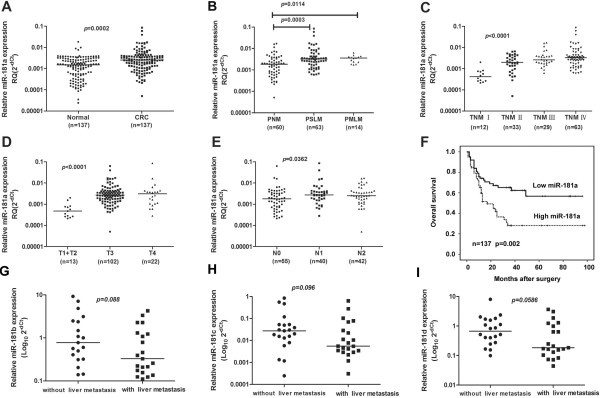
**Association between miR-181a expression in tumors and overall survival in CRC using qRT-PCR. (A)** The levels of miR-181a in 137 CRC tissues (Validation Cohort 1) were significantly higher than those in matched adjacent normal tissues; **(B)** The levels of miR-181a were significantly higher in patients with synchronous (PSLM) and metachronous liver metastasis (PMLM) than those without liver metastasis (PNM); **(C-E)** The expression levels of miR-181a were positively correlated with advanced TNM stages, T stage and N stage. Expression of miR-181a was presented in log10 scale and was normalised against an endogenous reference U6; **(F)** Kaplan-Meier overall survival curves for CRC patients were compared according to miR-181a expression level. Patients with higher miR-181a expression displayed a shorter overall survival after surgery. **(G-I)** The expressions of miR-181b-d in 21 paired CRC patients with and without liver metastasis by qPCR.

qRT-PCR analysis revealed a significant increase in miR-181a expression in tumor tissue (n = 137) compared with matched adjacent normal tissues (n = 137, fold change: 2.46, *p =* 0.0002). We then analyzed the relationship between miR-181a expression and liver metastasis. The relative expression ratio of miR-181a to U6 in patients with synchronous liver metastasis (n = 63) is significantly higher than those without metastasis (n = 60, fold change: 2.56, *p* = 0.0003). In addition, its expression in patients with metachronous liver metastasis (n = 14) is remarkably higher than those without metastasis (fold change: 1.93, *p* = 0.0114). These data demonstrate that miR-181a expression is elevated in CRC tumor tissues compared to surrounding normal tissue, and this elevated expression level of miR-181a correlates with liver metastasis in CRC patients (Figure 1).

Given that miR-181a is part of a family that includes miR-181a-d, we also checked the expression of three other miR-181 family members, miR-181b, miR-181c and miR-181d, to see whether they were also associated with metastases. Our results show that miR-181b-d expressions are not associated with CRC liver metastasis (Figure 1G-I). These results suggest that the association of miR-181a with CRC metastases is specific. The miRBase accession number and the mature sequence of the microRNAs studied are available in Additional file 1: Table S2.

### Elevated miR-181a expression correlates with advanced clinical stage, distant metastasis, and serves as an independent prognosis factor of poor survival

The clinical significance of miR-181a up regulation in CRC was further investigated in Validation Cohort 1. The expression of miR-181a increased remarkably with the advanced CRC TNM stages. Elevated miR-181a expression was significantly correlated with local tumor invasion (T stage), lymph node metastases or liver metastases, but not with other clinical-pathological parameters (Table 1, Figure 1C-E). Kaplan-Meier analysis showed that high miR-181a expression (above the median value in this 137 patient cohort) was associated with poor overall survival in CRC (Figure 1F). These results suggested that the up regulation of miR-181a may play an important role in the development and progression of CRC.

**Table 1:** Relationship between miR-181a expression and pathological features in CRC patients

	**miR-181a expression (RQ: 2 − ΔCt)**			
**Variable**	**Case no.**	**Median**	**Range**	**p**
Gender				0.646
Male	77	0.002267	0.00028409-0.086144702	
Female	60	0.002338	0.000051016-0.016974115	
Age				0.118
≤60(median)	64	0.0026165970	0.000051016-0.040007408	
>60	73	0.0018319950	0.000215355-0.086144702	
Venous invasion				0.18
Positive	50	0.002491	0.000473623-0.086144702	
Negative	87	0.002013	0.000051016-0.017423945	
T stage				< 0.0001
T1	1	0.0003546		
T2	12	0.000522	0.000215-0.002013	
T3	102	0.002697	0.000051-0.0635	
T4	22	0.003080	0.000286-0.08614	
N stage				0.0362
N0	55	0.001813	0.000215-0.0635	
N1	40	0.002767	0.000286-0.08615	
N2	42	0.002537	0.000051-0.01697	
M stage				0.0023
M0	74	0.002008	0.000051-0.01697	
M1	63	0.003277	0.000606-0.08615	
TNM stage				< 0.0001
I	12	0.0004145	0.000215-0.002013	
П	33	0.002306	0.000421-0.01586	
III	29	0.002267	0.000051-0.01697	
IV	63	0.002653	0.000353-0.08615	
Differentiation				0.4114
Poor	32	0.002538	0.000051-0.01577	
Moderate	76	0.002425	0.000266-0.08615	
Well	29	0.001738	0.000233-0.04001	
Histological type				
Adenocarcinoma	130	0.002339	0.000215-0.08615	0.5348
Mucinous adenocarcinoma	7	0.001962	0.000051-0.006384	

Quantified by stem–loop-based qRT–PCR. miR-181a was normalized to U6. Mann–Whitney test for the comparison between two groups or Kruskal–Walis test for more groups.

Furthermore, multivariate analysis was performed to determine the prognostic value of miR-181a expression using the Cox proportional hazard model. The risk variables examined included miR-181a expression level, as well as factors known to significantly affect the outcome of CRC such as age and gender of patients, differentiation, lymph node metastasis, surgical-pathological staging, histological type and venous invasion. In the univariate analysis, high miR-181a expression level in tumors (HR 2.22; 95% CI 1.307 to 3.77; *p* = 0.003), TNM staging (HR 11.057; 95% CI 3.995 to 30.605; *p* < 0.0001), venous invasion (HR 2.462; 95% CI 1.476 to 4.108; *p* = 0.001), and lymph node metastasis (HR 4.27; 95% CI 2.156 to 8.454; *p* < 0.0001) were significantly associated with survival; while age, gender, differentiation and histological type were not (Table 2). In the final multivariate Cox regression model, high miR-181a expression in tumors was associated with a poor survival prognosis (HR 1.871; 95% CI 1.078 to 3.246; *p* = 0.026) independent of other clinical covariates (Table 2).

**Table 2:** Univariate and multivariate Cox regression analysis of miR-181a expression levels and overall cancer survival in subjects with colorectal cancer

	**Cohort 1**				**Cohort 2**			
**Characteristic**	**Univariate analysis**		**Multivariate analysis**		**Univariate analysis**		**Multivariate analysis**	
	**HR (95% CI)**	**P Value**	**HR (95% CI)**	**PValue**	**HR (95% CI)**	**PValue**	**HR (95% CI)**	**P Value**
miR-181a expression	2.22 (1.307-3.77)	0.003	1.871 (1.078-3.246)	0.026	1.514 (1.223-1.874)	<0.001	1.382 (1.11-1.721)	0.004
High								
Low								
Gender	0.714 (0.423-1.206)	0.208	1.119 (0.650-1.927)	0.685	1.141 (0.845-1.54)	0.39	1.001 (0.734-1.366)	0.995
Male								
Female								
Age	1.034 (0.621-1.723)	0.898	1.023 (0.600-1.743)		1.075 (0.795-1.453)	0.637	1.147 (0.839-1.569)	0.389
≤60 (median)				0.935				
>60								
Venous invasion	2.462 (1.476-4.108)	0.001	1.243 (0.709-2.179)	0.447	2.375 (1.758-3.21)	<0.001	1.593 (1.15-2.207)	0.005
Positive								
Negative								
TNM stage	11.057 (3.995-30.605)	<0.0001	30.732 (8.165-115.679)	<0.0001	6.509 (4.02-10.54)	<0.001	8.363 (4.414-15.846)	<0.001
I-П								
III- IV								
Histological type	0.927 (0.290-2.966)	0.899	0.550 (0.148-2.043)	0.372	0.913 (0.428-1.945)	0.813	0.806 (0.354-1.836)	0.608
Adenocarcinoma								
Mucinous adenocarcinoma								
Lymph node metastasis	4.27 (2.156-8.454)	<0.0001	3.578 (1.417-9.033)	0.007	2.916 (2.062-4.125)	<0.001	1.686 (1.075-2.645)	0.023
Positive								
Negative								
Differentiation	0.629 (0.354-1.12)	0.115	0.532 (0.277-1.024)	0.059	0.438 (0.224-0.858)	0.016	0.912 (0.433-1.919)	0.808
Poor								
well								

### miR-181a localizes in colorectal epithelial cells and serves as an independent prognostic marker of CRC overall survival

**Figure 2:**
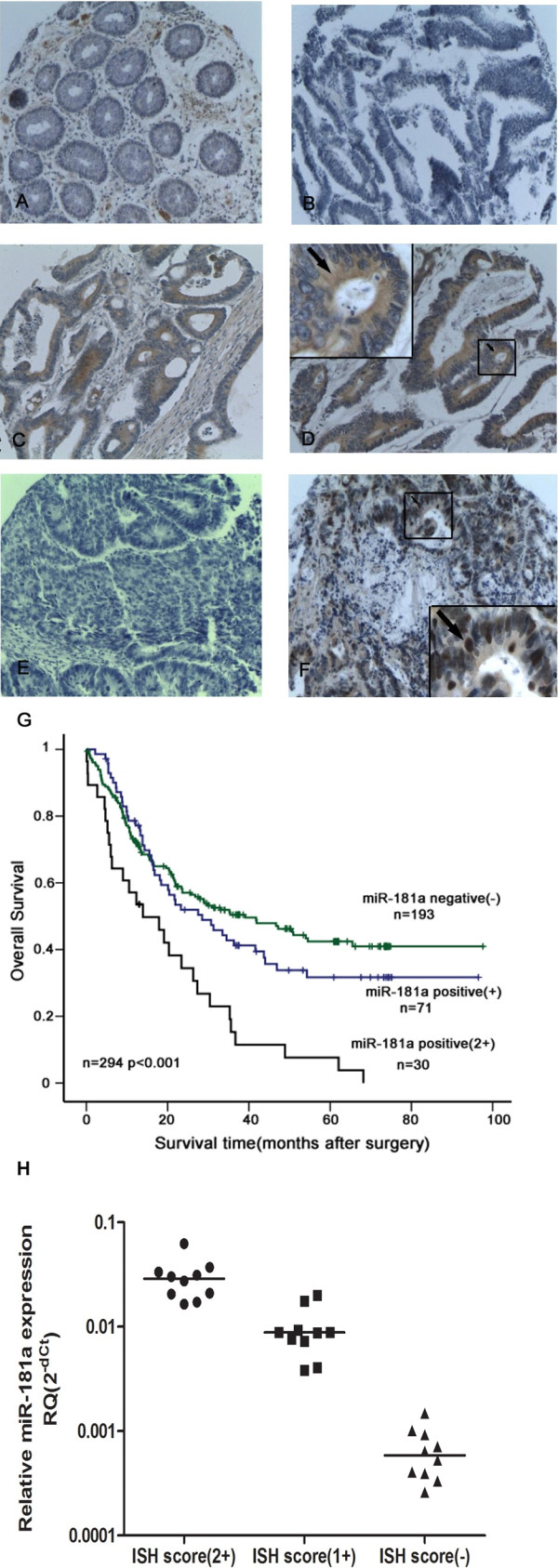
**miRNA detection in colorectal cancer tissues by LNA-ISH.** In situ hybridization analyses using 5′ DIG-labeled, LNA-modified DNA probes complementary to miR-181a, U6 small nuclear RNA and scrambled oligonucleotide (negative control) were performed on tissue microarray sections. **(A)** The expression of miR-181a was negative or weak in most of the normal colorectal mucosa; **(B)** Negative expression of miR-181a was also detected in some colorectal cancer tissues; **(C)** The weak positive expression of miR-181a was detected in the cytoplasm of cancer cells (+); **(D)** The strong positive expression of miR-181a was detected in the cytoplasm of cancer cells (2+); **(E)** No signal was detected in colorectal cancer tissues by using scrambled oligonucleotide probe (negative control); **(F) **The nuclear expression of U6 (positive control); **(G)** Association between miR-181a expression in tumors and overall survival in 294 patients with CRC using in situ hybridisation (cohort 2). Kaplan-Meier overall survival curves for CRC patients with follow-up information were compared according to miR-181a expression level. Patients with higher miR-181a expression displayed a shorter overall survival after surgery. **(H)** Association between miR-181a expression in CRC tumors and miR-181a ISH score. The levels of miR-181a detected by qPCR in 30 sample tissues that are matched to the samples used for ISH.

To further elucidate the functional role of miR-181a in CRC, we utilized in situ hybridization (ISH) to study the expression pattern of miR-181a in tumor and corresponding non-tumor tissue. A high level expression of miR-181a was detected in epithelial cells with elevated expression in tumor tissue compared with normal tissue. In cancerous tissue, miR-181a expression was detected only in the cytoplasm of tumor cells but not in tumor stroma, consistent with its role in tumor cells during colorectal carcinogenesis. Low levels of miR-181a expression were also seen in normal mucosa (Figure 2). To assess whether miR-181a expression is correlated with miR-181a ISH score, we assayed miR-181a expression by qPCR in 30 fresh CRC samples frozen in liquid nitrogen that were matched to the samples used for ISH. Our results showed that miR-181a expression was correlated with miR-181a ISH score (Figure 2H).

Expression of miR-181a was characterized in Validation Cohort 2, micro tissue arrays consisting of tumor samples from 294 CRC patients with different liver metastasis status, e.g., none, synchronous or metachronous liver metastasis to further validate the association between miR-181a expression and liver metastasis. Consistent with qRT-PCR results derived from Validation Cohort 1, miR-181a was significant less frequently expressed in PNLM (16/97, 16.5%) and normal tissue (14/72, 19.4%) than in PSLM (63/160, 39.4%) and PMLM (22/37, 59.5%) (*p* < 0.001)

Moreover, elevated miR-181a expression in tumor tissue detected by ISH was significantly associated with poor survival (*p* < 0.001) (Figure 2G). In univariate analysis of Validation Cohort 2, high expression of miR-181a in tumor tissue (HR 1.514; 95% CI 1.223 to 1.874; *p* < 0.001), TNM staging (HR 6.509; 95% CI 4.02 to 10.54; *p* < 0.001), venous invasion (HR 2.375; 95% CI 1.758 to 3.21; *p* < 0.001), differentiation (HR 0.438; 95% CI 0.224 to 0.858; *p* = 0.016) and lymphnode metastasis (HR 2.916; 95% CI 2.062 to 4.125; *p* < 0.001) were significantly associated with survival; while age, gender, and histological type were not. In the final multivariate Cox regression model, high miR-181a expression in tumor tissue was associated with a poor survival prognosis (HR 1.382; 95% CI 1.11 to 1.72; *p* = 0.004) independent of other clinical covariates consistent with results derived from Validation Cohort 1 (Table 2).

### miR-181a promotes CRC cell growth, invasion and liver metastasis

**Figure 3:**
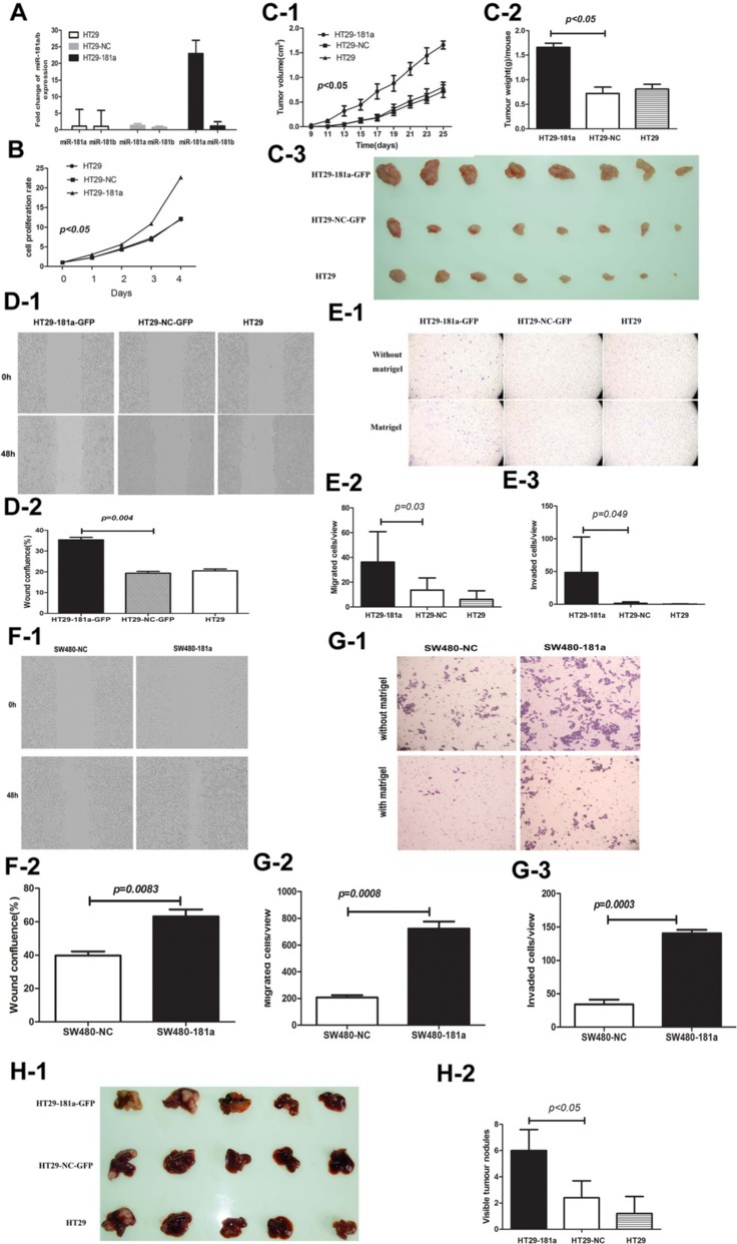
**Ectopic expression of miR-181a enhanced CRC cell *in vitro *migration and invasion as well as *in vivo *liver metastasis. (A)** The expression of miR-181a/b normalized to U6 was assayed in pri-miR-181a lentivirus infected HT-29 cells (HT29-181a), HT29 cells infected with control vector (HT29-NC) and parental HT29 cells (HT29). The data represent fold changes in miRNA expression. The limits of the 95% confidence intervals indicate variability of the changes; **(B)** Cell growth rates were stimulated by miR-181a in HT29 cells detected by CCK-8 assay. **(C)** The effect of miR-181a on the growth of HT-29 cells was evaluated in NOD-SCID mice. **(C-1)** Tumour growth curves. Points and bars represent the mean tumor volume with SD, eight mice in each group, p < 0.05; **(C-2)** Tumor weight derived from HT-29 cells expression different levels of miR-181a; **(C-3)** Tumors derived from HT-29 cells expression different levels of miR-181a; **(D)** Wound-healing assay shows that cell motility was enhanced following miR-181a over-expression in HT29 cells. **D-1**) Representative images from wounds made using a confluent monolayer of HT-29, HT29-NC and HT29-181a cells taken at 0 h and 48 h post wounding, imaged using IncuCyte ZOOM. **D-2**) The quantification results of cell motility (wound confluence) are plotted. Data represent the mean ± SD of three independent experiments. **(E)** Boyden chamber assays were performed with and without matrigel. The quantification results of migrated cells and invaded cells through Matrigel are plotted in **(E-2)** and **(E-3)**, respectively. Data represent the mean ± SD of three independent experiments with six random fields counted for each chamber; **(F)** Wound-healing assay shows that cell motility was enhanced following miR-181a over-expression in SW480 cells. **(G)** Cell migration and invasion were significantly enhanced in SW480 overexpressing miR-181a cells. **(H)** miR-181a enhances HT-29 cells liver metastasis. Representative livers derived from NOD-SCID mice are shown.

To explore the potential mechanism of miR-181a in promoting CRC progression, HT29 CRC cells were infected with pri-miR-181a lentivirus to determine its effects on cell growth *in vitro* and *in vivo*. Stable expression of miR-181a was confirmed using quantitative PCR (Figure 3A). Elevated miR-181a expression results in stimulated cell growth in culture (Figure 3B). Consistent with in-vitro results, the tumor growth curves showed that the growth of lentiviral miR-181a HT29 tumors was markedly increased compared with tumors derived from parental cell line HT-29, and lentiviral empty vector-infected cells (HT29-NC). Elevated miR-181a expression in HT29 CRC cells stimulated tumor growth in NOD-SCID mice resulting in increased tumor weight 1.66 ± 0.08 g (HT29-181a), 0.72 ± 0.13 (HT29-NC), 0.81 ± 0.10 g (HT29) (*p* < 0.05) (Figure 3C).

The effect of miR-181a on tumor invasion and metastasis was assessed. Cell wound healing assay showed that elevated miR-181a expression significantly enhanced tumor cell mobility in HT29-181a cells compared with HT29-NC or the parental HT 29 cells (*p =* 0.004) (Figure 3D). Furthermore, the ability of cell migration and cell invasion through matrigel were significantly enhanced by overexpressing miR-181a in HT29 cells (HT29-181a) compared with controls (HT29-NC and HT29) as demonstrated in Boyden Chamber assays (migration 2.74-fold, *p =* 0.03, invasion 34.7-fold, *p* = 0.049 respectively) (Figure 3E). The migration and invasion through matrigel of lenti–miR-181a-infected SW480 cells were dramatically enhance by 3.75-fold (*p =* 0.0008) and 4.67-fold (*p* = 0.0003) respectively, compared with controls (Figure 3G). A wound-healing assay revealed that the spreading of lenti–miR-181a-infected SW480 cells into the wound was much faster than the control cells (*p =* 0.0083) (Figure 3F). These results indicate that miR-181a stimulates colorectal cancer cell spreading, migration and invasion, suggesting its potential role in cancer metastasis.

The promoting effect of miR-181a on liver metastasis was demonstrated applying an experimental metastasis model by injecting HT29 overexpressing miR-181a cells into mouse spleens. HT29 cells with elevated miR-181a expression resulted in increased number of liver metastatic nodules as compared to that induced by HT29 cells transfected with control vector or the parental HT29 cells with average liver surface metastatic nodules of 6 ± 1.6; 2.4 ± 1.3; 1.2 ± 1.3 for HT29-181a, HT29-NC, and HT29 respectively (*p <* 0.05) (Figure 3H).

These combined *in vitro* and *in vivo* results illustrate the role of miR-181a in promoting tumor metastasis consistent with its clinical association with liver metastases in CRC patients.

### miR-181a targets the 3′-UTR of tumor suppressor gene WIF-1

**Figure 4:**
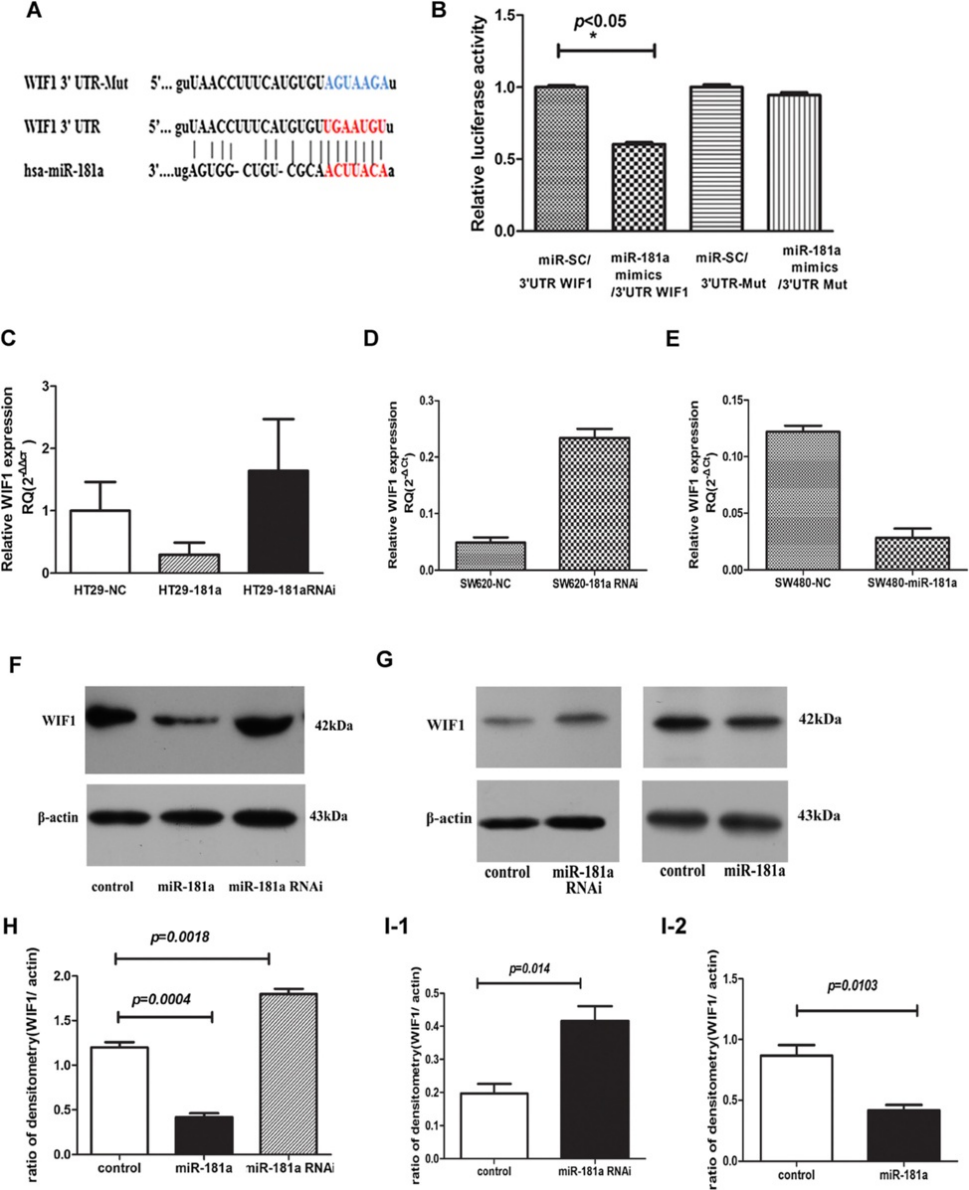
**WIF-1 is target of miR-181a. (A)** Schematic illustration of the predicted miR-181a-binding sites in WIF-1 3′-UTR; **(B)** Luciferase reporter assay demonstrates that miR-181a inhibited the wild-type, but not the mutant, 3′-UTRs of WIF-1 reporter activities compared with the vector alone control. The data represent the mean ± SD of three independent experiments with quadruplicates of sample. Student’ s t-test, * p < 0.05 versus control (wild-type 3 -UTR reporter vector + miR scramble) or mutant 3-UTR reporter group (mutant 3 -UTR reporter + miR-181a mimics/miR scramble); **(C)** The expression of endogenous WIF-1 was inhibited in the pool of lenti-pri-181a-infected HT29 cells and enhanced in lenti-pri-181a-RNAi-infected HT29 cells, compared with the control, at mRNA level as detected by qRT-PCR; bar, mRNA expression normalized to GAPDH mRNA; **(D, E)** WIF-1 mRNA levels were substantially enhanced in lenti-pri-181a-RNAi-infected SW620 **(D)** and suppressed in SW480 overexpressing miR-181a cells**(E)**, compared with the controls; **(F, G)** Western blot results show that the proteins of WIF-1 were down-regulated following lenti–pri–181a infection and up-regulated following lenti-pri-181a-RNAi infection (**F**, HT29 cell; **G**, SW620 and SW480cells). β-Actin served as an internal loading control. **(H, I)** The statistical analysis results of ratio of WIF1 compared to β-actin (**H**, HT29 cell; **I-1**, SW620; **I-2**, SW480 cells). Data represent the mean ± SD of three independent experiments.

To elucidate the biological mechanisms underlying the role of miR-181a in promoting tumor cell growth and metastasis, we investigated the potential targets of miR-181a. Target prediction programs, miRanda and TargetScan, were applied to identify WIF-1 as putative miR-181a target. The 3′-UTR of WIF-1 mRNA contains a complementary site for the seed region of miR-181a (Figure 4A). To verify this finding, WIF-1 3′-UTRs and its mutant containing the putative miR-181a binding sites were cloned downstream of the luciferase open reading frame. These reporter constructs were co-transfected into HEK293T cells with either miR-SC or miR-181a mimics. Increased expression of miR-181a upon infection of miR-181a mimics, significantly suppressed luciferase expression derived from reporter constructs containing wild type WIF-1 3′-UTRs with inhibition rates 40% (*p* < 0.05) comparing to cells co-transfected with miR-SC. This suppressive effect was abolished when mutated 3′-UTR of WIF-1 mRNAs, in which the binding sites for miR-181a were inactivated by site-directed mutagenesis, were co-infected with miR-181a (Figure 4B). These results support WIF-1 as putative target of miR-181a.

Functional regulation of WIF-1 expression by miR-181a was further analyzed by modulating miR-181a levels via overexpression or knockdown in three CRC cell lines, HT29, SW480 and SW620. WIF-1 mRNA levels were substantially suppressed in HT29 overexpressing miR-181a and SW480 overexpressing miR-181a cells as compared with that in control cells (Figure 4C, E). Meanwhile, the protein levels of WIF-1 were also suppressed after ectopic overexpression of miR-181a in HT29 and SW480 cell lines (Figure 4F, G). On the other hand, knock down of miR-181a via RNA interference in HT29 andSW620 cells resulted in increased mRNA and protein levels of WIF-1 (Figure 4C-G).

Collectively, these data support the bioinformatics prediction of WIF-1 as direct targets of miR-181a and established a functional association.

### Knockdown of miR-181a suppresses tumour growth and metastasis

**Figure 5:**
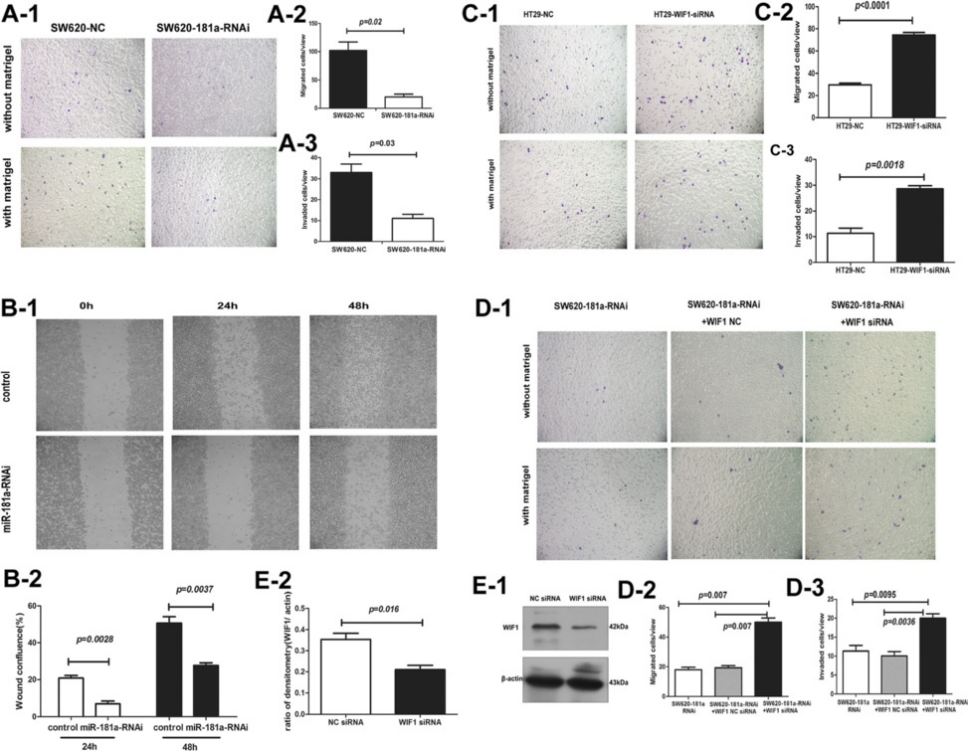
**Knockdown of miR-181a suppresses SW620 cell *in vitro *migration and invasion.** Prometastasis effect of miR-181a is mediated by inhibiting target genes WIF-1. **(A-1)** Cell migration and invasion through matrigel were significantly suppressed in miR-181a knockdown SW620 cells compared with the controls, as demonstrated by a Boyden chamber assay without matrigel or with matrigel. **(A-2, A-3)**The quantification results of migrated cells and invaded cells through matrigel are plotted in **(A-2)** and **(A-3)**, respectively. Results are displayed as the mean ± SD of three independent experiments with six random fields counted for each chamber. ∗Student’s *t*-test. **(B-1)** Wound-healing assay shows that cell motility was inhibited following miR-181a knockdown. **(B-2)** The quantification results of cell motility (wound confluence) are plotted. Data represent the mean ± SD of three independent experiments. **(C)** Knockdown of WIF-1 by siRNA in HT29 cells significantly promoted cell migration and invasion through matrigel. Representative fields of migration cells and invaded cells through matrigel on the membrane **(C-1)**. Average number of migration cells **(C-2)** and invaded cells **(C-3)** number per field from three independent experiments ± standard error (right). **(D-E)** SW620-181a-RNAi cells transfected with WIF-1 siRNA or a negative control (NC) siRNA. **(D-1, D-2, D-3)** Cell migration and invasion through matrigel assay with WIF-1 siRNA reversing miR-181a knockdown-mediated suppression of cell migration and invasion. **(E-1)** Western blot analysis for WIF-1 showing levels of WIF-1. **(E-2)** The statistical analysis results of ratio of WIF1 compared to β-actin. Data represent the mean ± SD of three independent experiments.

To confirm further the tumour-promoting function of miR-181a, we infected the highly metastatic cell line SW620 with miR-181a-RNAi lentivirus and measured its effects on cell spreading, migration and invasion *in vitro*. Consistent with the results of over-expressing miR-181a in HT-29 cells, the inhibition of miR-181a in SW620 cells remarkably decreased the cell motility (*p* = 0.02) and cell invasion through matrigel (*p* = 0.03), as demonstrated by Boyden chamber assays (Figure 5A). A wound-healing assay revealed that the spreading of lenti–miR-181a-RNAi infected SW620 cells into the wound was much slower than the control cells (Figure 5B).

### Knockdown of WIF-1 enhances tumour growth and metastasis

To explore the biological functions of WIF-1 in CRC cells, we determined whether inhibition of WIF-1 promoted CRC cell migration and invasion. In HT-29 cells, in vitro knockdown of WIF-1 promoted cell migration (*p <* 0.0001) and cell invasion (*p* = 0.0018) (Figure 5C). To further test this, we transfected SW620-181a-RNAi cells with siRNA for WIF-1 mRNA and found that the effect of miR-181a RNAi was partially attenuated by siRNA for WIF-1 mRNA (Figure 5D-E). These data confirmed that the prometastasis effect of miR-181a was mediated by inhibiting target genes WIF-1.

### miR-181a levels are inversely correlated with mRNA and protein expression of WIF-1 in CRC tissues

**Figure 6:**
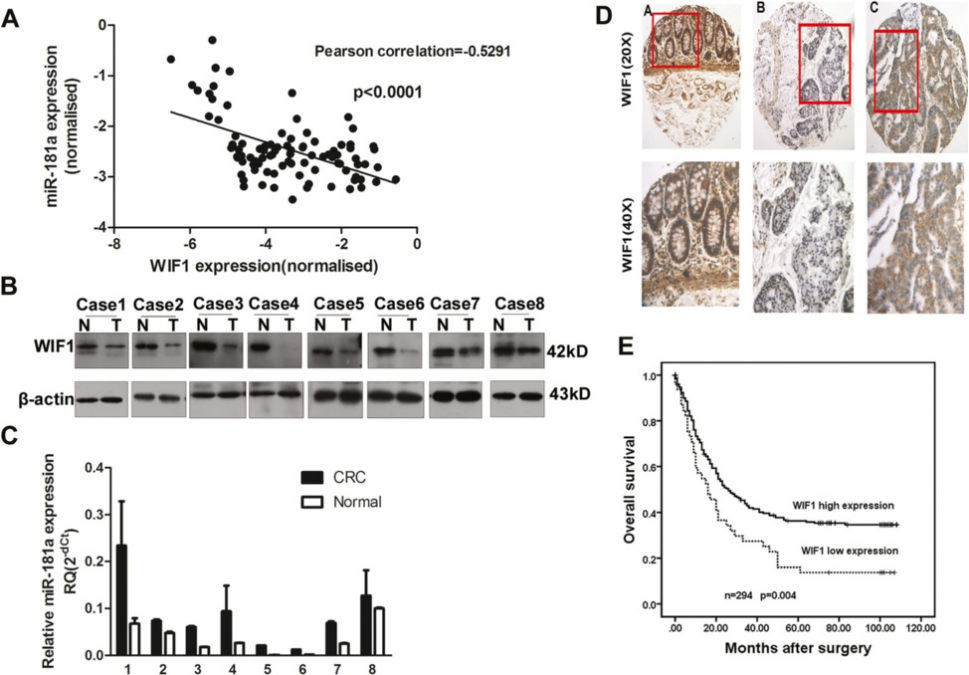
**miR-181a levels are inversely correlated with WIF-1 expression in CRC tissues. (A)** The relationship between miR-181a expression and WIF-1 mRNA levels in CRC samples (90 cases). Statistically significant reverse correlations were observed between miR-181a and WIF-1 mRNA levels with the use of two-tailed Spearman’s test; **(B)** Western blot results show that the proteins expression level of WIF-1 is lower in tumor tissue (T ) than normal adjacent tissues (N). **(C)** The levels of miR-181a in above 8 pairs of sample tissues. **(D)** Expression patterns of WIF-1 immunohistochemistry in tissue microarrays of Cohort 2. The expression of WIF1 proteins in adjacent non-malignant mucosa **(A)**, and CRC tissues with low **(B)** and high **(C)** WIF1 protein expression. Positive cells are stained brown. **(E)** Association between WIF1 expression in tumors and overall survival in 294 patients with CRC using immunohistochemistry (cohort 2). Kaplan-Meier overall survival curves for CRC patients with follow-up information were compared according to WIF1 expression level. Patients with lower WIF1 expression displayed a shorter overall survival after surgery.

The regulation of WIF-1 mRNA expression by miR-181a was further characterized in Validation Cohort 1 of 137 primary CRC tissues using qRT-PCR. A significant inverse correlation between the levels of miR-181a and mRNA expression of WIF-1 was confirmed with Pearson correlation = −0.5291; and *p* < 0.0001 (Figure 6A).

The protein expression of WIF-1 was also examined in paired CRC and normal tissues by Western Blot analysis. A significant decrease in WIF-1 expression was seen in tumor tissues compared with the matched adjacent normal tissue (Figure 6B), while their miR-181a expressions were shown an inverse trend (Figure 6C). The same inverse correlation was further demonstrated when protein expression of WIF-1 was examined in Validation Cohort 2 of TMA. High levels of miR-181a in tumor cells correlated with low levels of WIF-1 protein expression. Conversely, low levels of miR-181a in adjacent normal tissue correlated with high levels of WIF-1 protein expression (Figure 6D). We also investigated the prognostic significance of WIF-1 in CRC Cohort 2, Kaplan-Meier analysis was performed illustrating that low WIF-1 expression was associated with poorer overall survival in the CRC cohort 2(*p* = 0.004, Figure 6E).

### Overexpression of miR-181a induces epithelial-mesenchymal transition (EMT) in CRC

**Figure 7:**
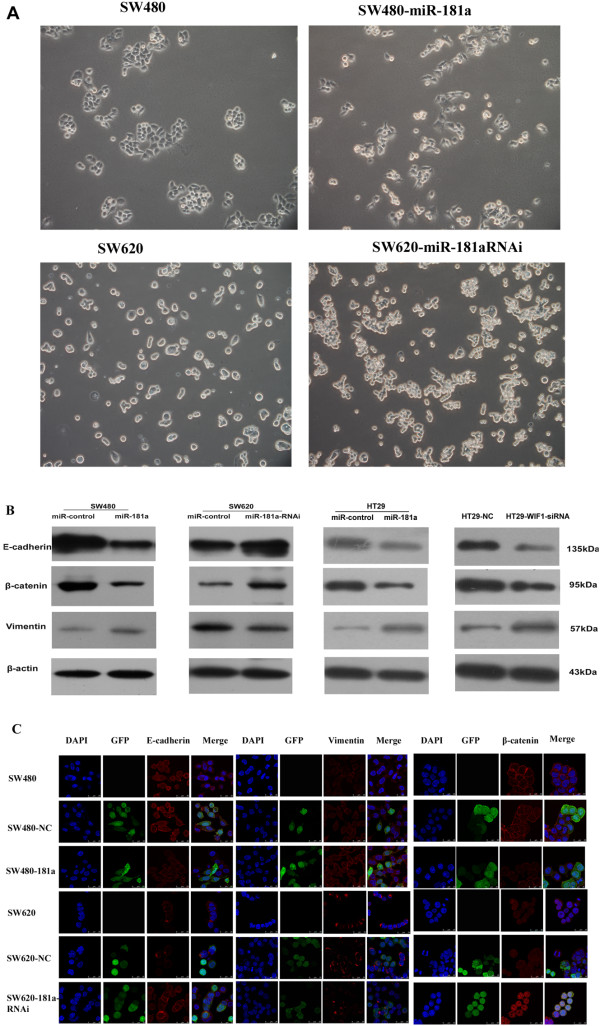
**Overexpression of miR-181a induces CRC cell epithelial mesenchymal transition. (A)** The morphology of SW480 and SW620 CRC cells with miR-181a overexpression or knockdown. The cell-to-cell contact was reduced in hsa-miR-181a–overexpressing SW480 cells and increased in miR-181a knockdown SW620 cells compared with the controls. **(B)** Expression of epithelial and mesenchymal markers were compared by Western blot analysis between miR-control-SW480 and miR-181a-SW480 cells, between miR-control-SW620 and miR-181a-RNAi-SW620 cells, between miR-control-HT29 and miR-181a-HT29 cells, between HT29-NC and HT29-WIF1-siRNA cells. β-actin was used as a loading control; **(C)** Immunofluorescence was used to compare the expression levels and pattern of epithelial and mesenchymal markers between miR-control-SW480 and miR-181a-SW480 cells, between miR-control-SW620 and miR-181a-RNAi-SW620 cells. Epithelial markers (E-cadherinand β-catenin, red signal) were down-regulated in miR-181a-SW480 cells and up-regulated in miR-181a-RNAi-SW620 cells; mesenchymal markers (vimentin, red signal) were up-regulated in miR-181a-SW480 cells and down-regulated in miR-181a-RNAi-SW620 cells.

The cell morphology of the stable SW480 hsa-miR-181a–overexpressing cell line was significantly altered; the cells had long and thin processes, cell-to-cell contact was reduced. The cell morphology of the miR-181a-knockdown SW620 was also altered, and the cell-to-cell contaction was increased (Figure 7A).

When miR-181a was overexpressed in SW480 cells, two epithelial markers, E-cadherin and β-catenin, were suppressed; while the mesenchymal marker, vimentin, was induced. On the other hand, knockdown of miR-181a in SW620 cells induced E-cadherin and β-catenin expression and suppressed vimentin expression (Figure 7B). Consistent results were also obtained when these CRC cells expressing different levels of miR-181a were examined with immunofluorescence staining (Figure 7C). We also assessed the epithelial and mesenchymal markers in HT29 overexpressing miR-181a cells. The results are consistent with results derived from SW480 cells (Figure 7B). We examined the effect of WIF1 on EMT in HT29 cell lines. The expression levels of E-cadherin and β-catenin were decreased; while vimentin was increased in HT29-WIF-1 siRNA cells (Figure 7B).

Taken together, these results suggest that elevated miR-181a levels in CRC may be involved in inducing EMT and therefore further promoting invasion and metastasis.

## Discussion

Recent studies have demonstrated that miRNA play important roles in tumor invasion and metastasis. In this study, miRNA profiles between five colorectal cancer tissues with synchronous liver metastases and five colorectal cancer tissues without liver metastases were compared. miR-181a demonstrated overexpression in colorectal cancer with liver metastasis.

Following microarray, we validated the deregulated miRNAs in two cohorts. We observed that up regulation of miR-181a is a frequent event in CRC tissues. High-level expression of miR-181a was significantly associated with colorectal cancer liver metastasis and especially with metachronous liver metastasis. Due to the largest absolute fold change, miR-181a was chosen for further study. The up regulation miR-181a was significantly associated with advanced clinical stage, distant metastasis and poorer overall survival in CRC.

Our results are similar with Nishimura’s report [9]. It is reported that high miR-181a expression group had a significantly poorer prognosis. High miR-181a expression was an independent significant prognostic factor for CRC. However, in Nishimura’s report, they did not find miR-181a to be differentially expressed between the normal colon and colorectal cancer tissue, nor did they see any associations between miR-181a and any clinicopathological parameters investigated. In the present study, qRT-PCR and ISH analysis revealed a significant increase in miR-181a expression in tumor tissue compared with matched adjacent normal tissues. We think the different result is associated with the different normal tissues origin and number in two studies. We think the difference of sample cohort’scomposition, especially the clinical stage distribution and the proportion of liver metastasis samples results in different analysis in the correlations between miR-181a expression and clinicopathological parameters.

The role of miR-181a in cancer development is complicated. For instance, some studies demonstrated that it is up-regulated in pancreatic cancer and breast cancer [10,11]. However, different groups showed that miR-181a was down-regulated in gliomas [12] and aggressive CLL. [13] Several studies have shown that miR-181 family members were up-regulated in hepatocellular cancer stem cells [14,15].

In this study, the effects of miR-181a on CRC development and progression were also investigated by both in-vitro and in-vivo assays. Overexpression of miR-181a could effectively promote CRC cell growth rate, migration and invasion in vitro and tumor growth and liver metastasis in vivo, whereas knockdown of miR-181a expression reduced CRC cell migration and invasion.

These findings suggest that miR-181a plays a critical role in the invasive and/or metastatic potential of CRC.

Each miRNA can potentially downregulate many target genes through binding their 3′-UTR. One important task is to identify the downstream target genes regulated by the dysregulated miRNA. In the present study, quantitative PCR and western blot analysis demonstrated that miR-181a could decrease the expression of WIF-1 in both mRNA and protein levels. In addition, inhibiting miR-181a expression could increase WIF-1 expression. Luciferase assay showed that miR-181a could interact with the 3′-UTR of WIF-1. We also found that knockdown of WIF-1 gene expression by siRNA reversed miR-181a-RNAi-mediated suppression of tumor cell migration. Moreover, the expression of WIF-1 was negatively correlated with miR-181a in clinical CRC specimens. These observations indicate that miR-181a mechanistically acts via the regulation of WIF-1. Our results also showed that low-level expression of WIF-1 is positively correlated with tumor metastasis and/or poor prognosis. This is consistent with our finding that up regulation of miR-181a is associated with CRC liver metastasis and poorer overall survival.

WIF-1, Wnt inhibitory factor-1, is a secreted antagonist that can bind to Wnt proteins directly and inhibit Wnt signaling pathway. It has been reported that WIF-1 expression is down regulated in several human cancer including prostate, bladder, breast, nonsmall-cell lung carcinomas and colorectal cancer [16-18]. Several groups have reported that restoring WIF-1 expression in cancer cells could inhibit cancer cell growth [19,20]. Our results, along with the roles of WIF-1 in tumor progression, indicate the involvement of the miR-181a-WIF-1 regulation chain in CRC progression.

It is reported that miR-181 directly targets nemo-like kinase (NLK), an inhibitor of wnt/β-catenin signaling in Hepatocellular cancer [15]. We speculate that miR-181a could affect colorectal cancer cells EMT. EMT appears to be a key event in tumor invasion and metastasis [21,22]. Thus, epithelial cells lose their epithelial adherence and tight junction proteins, lose their polarity and cell-cell contact, and undergo remarkable remodeling of the cytoskeleton, all of which facilitates cell motility and invasion. In present study, we found that the overexpression of miR-181a induces EMT, as shown by the decreased expression of the epithelial markers E-cadherin and β-catenin, and enhanced expression of the mesenchymal markers vimentin.

These findings suggest that miR-181a plays a critical role in regulating epithelial-emesenchymal cell transition and, ultimately, promotes the invasive and/or metastatic potential of CRC, probably by its direct target on WIF-1 gene. We further assessed whether the miR-181a induced EMT is due to the direct regulation of WIF1. We found that inhibition of WIF1 could lead to EMT in HT29 cell line, which suggesting that the miR-181a induced EMT is due to the direct regulation of WIF1.

In summary, we demonstrated in this study that the higher expression of miR-181a in CRC specimens is associated with liver metastasis and poor survival, and in particular, the higher expression of miR-181a is associated with metachronous liver metastasis. In this study, we also investigated the potential role of miR-181a in CRC liver metastasis and its underlying mechanisms. Our data suggest that up regulation of miR-181a plays an important role in CRC cell metastasis, and that the tumor-promoting function of miR-181a is through repressing its downstream target gene WIF-1. miR-181a could be employed as a new prognostic marker and/or as an effective therapeutic target for CRC.

## Materials and methods

### Cell line and experimental animals

Human CRC cell lines HT29, SW480 and SW620 were purchased from American Type Culture Collection and were maintained in Dulbecco’s Modified Eagle medium (Gibco, Carlsbad, CA) with 10% fetal bovine serum, 100 U/ml penicillin sodium and 100 mg/ml streptomycin sulfate in humidified 5% CO_2_ at 37°C. Male NOD-SCID mice (4–6 weeks old) were purchased from Beijing HFK Bio-technology Co. Ltd. (Beijing, China). Mice were maintained in a pathogen-free facility and used in accordance with the institutional guidelines for animal care.

### Patients and samples

CRC samples were collected from surgical resection at Peking University Cancer Hospital & Institute (Beijing, China). All samples were immediately frozen in liquid nitrogen and stored at −80°C or fixed in 10% formalin for paraffin embedding. Samples collection and usage in the present study were approved by the Ethics Review Committees of Peking University Cancer Hospital & Institute.

The Testing Cohort consisted of CRC tumor specimens from patients with synchronous liver metastases (n = 5) or without (n = 5) liver metastases (follow up > 6 years). Samples were analyzed using microarray to generate differential miRNA profiling between CRC tumor tissues with and without liver metastases. Validation Cohort 1 consisting of fresh CRC and surrounding non-tumor tissue were obtained from 137 patients who underwent surgical resections from 2001 to 2006. These samples were analyzed by quantitative reverse transcription PCR (qRT-PCR). Validation Cohort 2, independent from Validation Cohort 1, consisting of CRC and surrounding non-tumor tissues were obtained from 294 patients who underwent surgical resections from 1999 to 2006. These samples were paraffin-embedded and micro-dissected to generate tissue microarrays (TMAs) for in situ hybridization (ISH) and immunohistochemstry. All samples were collected from patients prior to receiving any preoperative chemotherapy or radiotherapy, and were verified to contain at least 80% tumor cells. Cases with familial adenomatous polyposis CRC were excluded from the study. Final cut off date of follow-up was January 30, 2012. All samples have follow-up data. A summary of the clinical characteristics of these patients is shown in Additional file 1: Table S3 and S4.

### RNA isolation and miRNA profiling

Total RNA was extracted from 5 CRC tissues with and 5 without liver metastases using TRIZOL reagent (Invitrogen) according to the manufacturer’s instructions. miRNA microarray profiling was performed at CapitalBio Corporation (Beijing, China) following standard procedures detailed on http://www.capitalbio.com. Fluorescein-labeled miRNA were used for hybridization on Mammalian miRNA arrayV2.0 (CapitalBio, Beijing, China) containing 743 probes in triplicate, corresponding to 576 human miRNAs (including 122 predicted miRNAs). Hybridization signals were detected and arrays were scanned with a LuxScanTM 10 K/A laser confocal scanner (CapitalBio, Beijing, China). The raw pixel intensities were extracted using the LuxScan3.0 software (CapitalBio, Beijing, China). The data were normalized between slides using the quantile normalization method proposed by Bolstad et al. [23]. The differentially expressed genes, classified as those with Fold changes above 2, were selected using the SAM software, version 2.1.

### Quantitative real-time RT-PCR (qRT-PCR)

Total RNA was extracted using miRNeasy Mini Kit (Qiagen, Hilden, Germany). For miRNA quantification, the miRNAs levels were assayed using TaqMan MicroRNA assays, according to the manufacturer’s protocol (Applied Biosystems, Carlsbad, CA). The u6 small nuclear B noncoding RNA (RNU6B, Applied Biosystems, Carlsbad, CA) level was used as an internal normalization control.

For mRNA detection, total RNA (2 μg) was used in 20 μl of reverse transcriptase reaction to synthesize cDNA by using Moloney murine leukemia virus reverse transcriptase (M-MLV RT) for RT-PCR (Invitrogen, Carlsbad, CA), according to the manufacturer’s protocol. Real-time PCR was performed using the above RT products with the SYBR Green PCR Master Mix (Toyobo Co. Ltd., Osaka, Japan) on an ABI7500 PCR machine. PCR cycling conditions were: 95°C for 1 minute, 40 cycles of 95°C for 15 seconds, 60°C for 15 seconds and 72°C for 45 seconds. Data are presented as relative quantification (RQ) to U6 or GAPDH based on calculations of 2^-ΔCt^ where ΔCt = C_t(Target)_-C_t(Reference)_, or as ΔCt. Fold changes were calculated by the 2^-ΔΔCt^. All primers are listed in Additional file 1: Table S5.

### Tissue microarrays

Paraffin-embedded samples were microdissected and tissue microarrays (TMAs) were constructed in quadruplicates using a previously described method [24]. Seven TMAs were constructed to include 160 Primary CRC tumors with Synchronous Liver Metastasis (PSLM), 97 Primary CRC tumors with Non-Liver Metastasis (PNLM), 37 Primary CRC tumors with metachronous liver metastasis (PMLM), and 72 paired adjacent normal colorectal mucosa and 32 matched liver metastasis tissues.

### In situ hybridization analysis

Digoxigenin (DIG)-labeled mercury locked nucleic acid probes for human miR-181a, U6 (positive control) and scramble DNA (negative control) were purchased from TaKaRa (Dalian, China). The in situ hybridization (ISH) was performed with a modified version of the protocol for formalin-fixed paraffin-embedded tissue by Wigard Kloosterman (http://www.exiqon.com/ls/Documents/Scientific/FFPE%20in%20situ%20hybridization.pdf) on human colorectal cancer tissues. The sequence and hybridization temperature of probes were showed in Additional file 1: Table S5.

The slides were then scored by two pathologists independently as negative (−), weak or focally positive (1+), or strongly positive (2+) [25]. Both pathologists were blinded to the patient’s clinical outcome.

### Oligonucleotide, lentiviral vector construction and cell infection

miRNA mimics, the miRNA inhibitor and negative control miRNA oligonucleotides of has-miR-181a were from RiboBio Co. Ltd (Guangzhou, China). The small interfering RNAs (siRNAs) targeting WIF-1 were synthesized by RiboBio Co. Ltd. An unrelated sequence was used as a negative control (provided by RiboBio). The sequence was WIF1 siRNA, 5′ GAGUACUCAUAGGAUUUGA dTdT -3′ (sense). Stable transfectants overexpressing or knockdown miR-181a were generated by lentiviral transduction using a pGCsil-GFP vector (GeneChem Co., Ltd, Shanghai,China). A lentiviral vector expressing green fluorescent protein alone (LV-GFP) was used as a control. Transfection of oligonucleotides or lentivirus construction was performed using the Lipofectamine 2000 reagent (Invitrogen) according to the manufacturer’s instructions. HT-29, SW480 and SW620 cells were infected with recombinant lentivirus-transducing units plus 8 μg/ml Polybrene (Sigma, St Louis, Missouri, USA).

### In vitro cell growth, spreading, motility and invasion assays

Cell growth was assessed using CCK-8 (Dojindo, Tokyo, Japan) according to the manufacturer’s instructions.

Cell spreading was evaluated by a wound healing assay. The wound healing assay was performed by the CellPlayer™ 96-Well Kinetic Cell Migration assay (The Essen BioScience IncuCyte^TM^ Live-Cell Imaging System). Cells were seeded into 96-well plate and cultured until attached. The woundMaker™ and wounding procedure were used to create precise and reproducible wounds in all wells of the 96-well plate. The plate was placed into the IncuCyte™ ZOOM to scan every hour for migration assays. The data was analyzed by wound confluence, Wound confluence (%) represents the fractional area of the wound that is occupied by cells.

Cell motility or invasion was measured using a Boyden chamber assay with or without Matrigel. Photographs of three randomly selected fields of the fixed cells were taken and cells were counted. Experiments were repeated three times independently. These assays were carried out as previously described [26].

### In vivo tumor growth and liver metastasis assay

For the tumorigenecity study, HT29 cells, has-miR-181a-expressing cells (HT29-181a+) and empty vector cells (HT29-NC) were injected subcutaneously into the back of NOD-SCID mice (8 mice/group). Over a 4-week period, tumor formation in mice was observed by measuring the tumor volume calculated by the formula V = 0.5xLxW^2^. Tumors were then excised and weighed.

HT29 cells, has-miR-181a-expressing cells (HT29-181a+) and empty vector cells (HT29-NC) were assayed for metastasis in 5- to 6-week-old NOD-SCID mice using the splenic injection model as described previously [27]. Cells (1 × 10^6^) were injected into the spleen subcapsular through an abdominal incision under sterile conditions. After injection, the spleen was returned gently back to abdominal cavity and homoeostasis was assured. The area was thoroughly irrigated with warm sterile water and the abdominal cavity was closed in appropriate layers. Ten weeks after surgery, mice were euthanized and the livers were obtained to determine the liver weight and hepatic metastatic foci.

All animal experiments were reviewed and approved by the Ethics Review Committee at the Peking University School of Oncology.

### Luciferase reporter assay

The 3′-UTR of WIF-1 was amplified via polymerase chain reaction (PCR) from human genomic DNA constructed and cloned into pmiR-RB-REPORT™Luciferase, downstream of the firefly luciferase gene to form pMIR-3′-UTR WIF-1 and pMIR-3′-UTR WIF-1-Mut constructs (primers are listed in Additional file 1: Table S3).

For the luciferase assay, HEK 293 T cells were seeded in 96-well plates, pMIR-3′-UTR constructs were co-transfected with miR-181a mimics or miR scramble (miR-SC) (50 nM, synthesized by Ribobio Co. Guangzhou, China) using the Lipofectamine2000 method. The activities of firefly luciferase and Renilla luciferase were measured at 24 h post-transfection using the dual-luciferase reporter assay kit (Promega) following the manufacturer’s protocol. Firefly luciferase activity was normalized to that of the Renilla luciferase for each sample. Each experimental group consisted of three wells and the experiment was repeated three times.

### Western blot analysis

Total protein lysates extracted from samples were quantitated using BCA protein assay kit (Pierce, Rockford, IL). 20 μg total protein lysates extracted from samples were separated with 10% sodium dodecyl sulfate-polyacryl-amide gels and transferred to a polyvinylidene fluoridemembrane. The membrane was blocked with 3% BSA, followed by incubation with mouse monoclonal anti-human WIF-1 (Abcam, Cambridge, MA), rabbit polyclonal anti-human E-cadherin, β-catenin and vimentin (Cell Signaling Technology Inc., Danvers, MA), anti-human β-Actin antibody (Epitomics, Burlingame, CA). The membrane was then incubated with secondary horseradish peroxidase-conjugated goat anti-rabbit or anti-mouse antibody (Jackson Immune Research Laboratories Inc., West Grove, PA), and visualized using Immobilon™ Western Chemiluminescent HRP substrate (Millipore). β-Actin was used as the loading control.

### Immunohistochemical analysis on Tissue Microarray (TMA)

Immunohistochemical studies of WIF-1 were done on tissue microarray using the Polymer Detection System for immune-histological staining (PV9000 kit, ZSJQ-Bio, China). All images were examined by two experienced pathologists independently. Immunostaining was evaluated as described [28] previous.

### Immunofluorescence analysis

SW480, SW480-NC, SW480-181a, SW620, SW620-NC, SW620-181a RNAi cells were plated in a 96-well microplate and were cultured overnight until cells were adherent. Cells were fixed and stained with anti-E-cadherin, −β-catenin and-vimentin primary antibody and DyLight549 Goat Anti-rabbit Secondary Antibody. Cells were also labeled with Hoechst 33342 Dye for nuclear staining. Expression of E-cadherin, β-catenin and vimentin was imaged using a laser scanning confocal microscopy (TCS-SP2, Leica Microsystems).

### Statistical analysis

All data are presented as the mean ± standard error (SE) and were analyzed using the statistical package SPSS13.0. Data are expressed as median values (interquartile range) in supplementary tables or as means ± SE in the figures. Comparisons were performed using the nonparametric Mann–Whitney U test or Kruskal-Walis test for continuous variables. Spearman’s rho correlation test (two tailed) was used to estimate the correlation between the expression in CRC tissue of each marker and clinical-pathological variables. Survival curves were plotted using the Kaplan-Meier analysis and the log-rank test was used to test for significant differences. Cox univariate and multivariate proportional hazard models were used to estimate the hazard ratio for each marker. *P* ≤ 0.05 was considered statistically significant.

## Competing interests

The authors declare that they have no competing interests.

## Authors’ contributions

D-BJ contributed to study design, data collection, analysis, and interpretation; Z-GC performed tissue microarrays and in situ hybridization analysis; ML carried out immunoassays; T-CZ contributed to validation of target gene; Y-FY contributed to statistical analysis; J-ZX, LY and Z-QZ contributed to the discussion of the results; JG outlined the experimental design of the study, contributed to the discussion of the results and revised the manuscript. All authors contributed to writing, critical review, and final approval to submit the paper for publication.

## Supplementary Material

Additional file 1: Table S1 All detected miRNAs expression in CRC patients without liver metastasis compared to patients with liver metastasis by microarray profiling screen. **Table S2**. The miRBase accession number and the mature sequence of the microRNA studied. **Table S3**. Clinical characteristics of colorectal cancer patients of Testing Cohort. **Table S4**. Clinical characteristics of colorectal cancer patients of Validation Cohort 1 and 2. **Table S5**. Primer sequences.” We highlight the paragraph in the resubmitted version. Click here for file
