# Supplementary material for: MicroRNA-181a promotes tumor growth and liver metastasis in colorectal cancer by targeting the tumor suppressor WIF-1
Source: Mol Cancer. 2014 Apr 23;13:86. doi: 10.1186/1476-4598-13-86 (PMC4021214; doi:10.1186/1476-4598-13-86)
Supplement: Additional file 1: Table S1 — All detected miRNAs expression in CRC patients without liver metastasis compared to patients with liver metastasis by microarray profiling screen. Table S2. The miRBase accession number and the mature sequence of the microRNA studied. Table S3. Clinical characteristics of colorectal cancer patients of Testing Cohort. Table S4. Clinical characteristics of colorectal cancer patients of Validation Cohort 1 and 2. Table S5. Primer sequences.” We highlight the paragraph in the resubmitted version. [file 1476-4598-13-86-S1.doc]

**Additional file 1**

**Table S1 All detected miRNAs expression in CRC patients without liver metastasis compared to patients with liver metastasis by microarray profiling screen**

| **Positive genes** | | | | | | | | | |
| --- | --- | --- | --- | --- | --- | --- | --- | --- | --- |
| **Gene ID** | **Gene Name** | | **Score(d)** | **Numerator(r)** | **Denominator(s+s0)** | **Fold Change** | **q-value(%)** | | **localfdr(%)** |
| hsa-let-7d | [hsa-let-7d](http://genome-www4.stanford.edu/cgi-bin/SMD/source/sourceResult?choice=Gene&option=Name&criteria=hsa-let-7d) | | 1.681480942 | 1.096753102 | 0.652254257 | 1.900560849 | 6.354166667 | | 26.44625676 |
| hsa-miR-15b | [hsa-miR-15b](http://genome-www4.stanford.edu/cgi-bin/SMD/source/sourceResult?choice=Gene&option=Name&criteria=hsa-miR-15b) | | 1.651125876 | 2.118436894 | 1.283025677 | 1.93390545 | 6.354166667 | | 26.92178549 |
| hsa-miR-16 | [hsa-miR-16](http://genome-www4.stanford.edu/cgi-bin/SMD/source/sourceResult?choice=Gene&option=Name&criteria=hsa-miR-16) | | 1.639029642 | 1.147906838 | 0.700357582 | 1.785952637 | 6.354166667 | | 27.11402067 |
| hsa-let-7f | [hsa-let-7f](http://genome-www4.stanford.edu/cgi-bin/SMD/source/sourceResult?choice=Gene&option=Name&criteria=hsa-let-7f) | | 1.618242392 | 1.234025552 | 0.762571515 | 1.841359905 | 6.354166667 | | 27.45186488 |
| hsa-miR-20b | [hsa-miR-20b](http://genome-www4.stanford.edu/cgi-bin/SMD/source/sourceResult?choice=Gene&option=Name&criteria=hsa-miR-20b) | | 1.589553641 | 1.594689662 | 1.003231109 | 1.913052563 | 6.354166667 | | 27.94205115 |
| hsa-let-7g | [hsa-let-7g](http://genome-www4.stanford.edu/cgi-bin/SMD/source/sourceResult?choice=Gene&option=Name&criteria=hsa-let-7g) | | 1.457151055 | 0.944736119 | 0.64834467 | 1.734752563 | 6.354166667 | | 31.12399011 |
| hsa-let-7a | [hsa-let-7a](http://genome-www4.stanford.edu/cgi-bin/SMD/source/sourceResult?choice=Gene&option=Name&criteria=hsa-let-7a) | | 1.421062662 | 0.873063646 | 0.614373784 | 1.729304183 | 6.354166667 | | 32.36353274 |
| hsa-miR-26b | [hsa-miR-26b](http://genome-www4.stanford.edu/cgi-bin/SMD/source/sourceResult?choice=Gene&option=Name&criteria=hsa-miR-26b) | | 1.393046856 | 1.013854992 | 0.727796763 | 1.583941505 | 6.354166667 | | 33.42883458 |
| hsa-miR-223 | [hsa-miR-223](http://genome-www4.stanford.edu/cgi-bin/SMD/source/sourceResult?choice=Gene&option=Name&criteria=hsa-miR-223) | | 1.392999067 | 0.832659336 | 0.597745796 | 2.018441975 | 6.354166667 | | 33.43071207 |
| hsa-miR-224 | [hsa-miR-224](http://genome-www4.stanford.edu/cgi-bin/SMD/source/sourceResult?choice=Gene&option=Name&criteria=hsa-miR-224) | | 1.390883747 | 1.498063023 | 1.0770584 | 1.767923044 | 6.354166667 | | 33.51399153 |
| hsa-let-7e | [hsa-let-7e](http://genome-www4.stanford.edu/cgi-bin/SMD/source/sourceResult?choice=Gene&option=Name&criteria=hsa-let-7e) | | 1.347913192 | 1.686553931 | 1.251233344 | 1.204745234 | 6.354166667 | | 35.26065277 |
| hsa-miR-193a | [hsa-miR-193a](http://genome-www4.stanford.edu/cgi-bin/SMD/source/sourceResult?choice=Gene&option=Name&criteria=hsa-miR-193a) | | 1.261132047 | 1.214857847 | 0.963307411 | 1.740179941 | 17.59615385 | | 38.87742745 |
| hsa-miR-451 | [hsa-miR-451](http://genome-www4.stanford.edu/cgi-bin/SMD/source/sourceResult?choice=Gene&option=Name&criteria=hsa-miR-451) | | 1.243762469 | 1.142252812 | 0.918385014 | 1.428740457 | 17.59615385 | | 39.59406676 |
| hsa-miR-18b | [hsa-miR-18b](http://genome-www4.stanford.edu/cgi-bin/SMD/source/sourceResult?choice=Gene&option=Name&criteria=hsa-miR-18b) | | 1.222826731 | 1.289073482 | 1.054175092 | 1.437982412 | 17.59615385 | | 40.44739302 |
| hsa-miR-424 | [hsa-miR-424](http://genome-www4.stanford.edu/cgi-bin/SMD/source/sourceResult?choice=Gene&option=Name&criteria=hsa-miR-424) | | 1.115756283 | 1.312008935 | 1.175892043 | 1.041959215 | 17.59615385 | | 44.54532085 |
| hsa-miR-15a | [hsa-miR-15a](http://genome-www4.stanford.edu/cgi-bin/SMD/source/sourceResult?choice=Gene&option=Name&criteria=hsa-miR-15a) | | 1.087135511 | 0.582789451 | 0.536078019 | 1.342793228 | 17.59615385 | | 45.61722615 |
| hsa-miR-107 | [hsa-miR-107](http://genome-www4.stanford.edu/cgi-bin/SMD/source/sourceResult?choice=Gene&option=Name&criteria=hsa-miR-107) | | 1.062237229 | 0.590051406 | 0.555479878 | 1.478770758 | 43.57142857 | | 46.57322444 |
| hsa-miR-335 | [hsa-miR-335](http://genome-www4.stanford.edu/cgi-bin/SMD/source/sourceResult?choice=Gene&option=Name&criteria=hsa-miR-335) | | 1.054349299 | 0.633202255 | 0.600562125 | 1.479447191 | 43.57142857 | | 46.88279117 |
| hsa-miR-101 | [hsa-miR-101](http://genome-www4.stanford.edu/cgi-bin/SMD/source/sourceResult?choice=Gene&option=Name&criteria=hsa-miR-101) | | 1.044960286 | 1.007447049 | 0.964100801 | 1.282474803 | 43.57142857 | | 47.25574949 |
| hsa-miR-199b | [hsa-miR-199b](http://genome-www4.stanford.edu/cgi-bin/SMD/source/sourceResult?choice=Gene&option=Name&criteria=hsa-miR-199b) | | 1.036081987 | 0.736081595 | 0.710447247 | 1.349394995 | 43.57142857 | | 47.61299898 |
| hsa-miR-130a | [hsa-miR-130a](http://genome-www4.stanford.edu/cgi-bin/SMD/source/sourceResult?choice=Gene&option=Name&criteria=hsa-miR-130a) | | 1.024515554 | 1.100705603 | 1.074366903 | 1.086493731 | 43.57142857 | | 48.08545544 |
| hsa-miR-30c | [hsa-miR-30c](http://genome-www4.stanford.edu/cgi-bin/SMD/source/sourceResult?choice=Gene&option=Name&criteria=hsa-miR-30c) | | 1.021374284 | 0.497291633 | 0.486884819 | 1.385839611 | 43.57142857 | | 48.21531084 |
| hsa-miR-28 | [hsa-miR-28](http://genome-www4.stanford.edu/cgi-bin/SMD/source/sourceResult?choice=Gene&option=Name&criteria=hsa-miR-28) | | 1.00259811 | 0.841155685 | 0.838975933 | 1.165620064 | 43.57142857 | | 49.00786613 |
| hsa-miR-203 | [hsa-miR-203](http://genome-www4.stanford.edu/cgi-bin/SMD/source/sourceResult?choice=Gene&option=Name&criteria=hsa-miR-203) | | 0.973571142 | 0.49171401 | 0.505062228 | 1.445057833 | 43.57142857 | | 50.30182419 |
| hsa-miR-199a* | [hsa-miR-199a*](http://genome-www4.stanford.edu/cgi-bin/SMD/source/sourceResult?choice=Gene&option=Name&criteria=hsa-miR-199a*) | | 0.950977502 | 0.563696652 | 0.592754982 | 1.376065804 | 43.57142857 | | 51.3820423 |
| hsa-miR-126 | [hsa-miR-126](http://genome-www4.stanford.edu/cgi-bin/SMD/source/sourceResult?choice=Gene&option=Name&criteria=hsa-miR-126) | | 0.949498966 | 0.584944161 | 0.616055606 | 1.325776342 | 43.57142857 | | 51.45520536 |
| hsa-miR-103 | [hsa-miR-103](http://genome-www4.stanford.edu/cgi-bin/SMD/source/sourceResult?choice=Gene&option=Name&criteria=hsa-miR-103) | | 0.937412523 | 0.53374011 | 0.569375912 | 1.480896115 | 43.57142857 | | 52.06468457 |
| hsa-miR-215 | [hsa-miR-215](http://genome-www4.stanford.edu/cgi-bin/SMD/source/sourceResult?choice=Gene&option=Name&criteria=hsa-miR-215) | | 0.930110623 | 1.311162127 | 1.409684068 | 1.17952511 | 43.57142857 | | 52.44271029 |
| hsa-miR-1 | [hsa-miR-1](http://genome-www4.stanford.edu/cgi-bin/SMD/source/sourceResult?choice=Gene&option=Name&criteria=hsa-miR-1) | | 0.900630076 | 1.278552674 | 1.419620228 | 0.797464597 | 43.57142857 | | 54.04360831 |
| hsa-miR-660 | [hsa-miR-660](http://genome-www4.stanford.edu/cgi-bin/SMD/source/sourceResult?choice=Gene&option=Name&criteria=hsa-miR-660) | | 0.89594177 | 0.630414255 | 0.703633067 | 1.862411785 | 43.57142857 | | 54.30916563 |
| hsa-miR-98 | [hsa-miR-98](http://genome-www4.stanford.edu/cgi-bin/SMD/source/sourceResult?choice=Gene&option=Name&criteria=hsa-miR-98) | | 0.878492311 | 1.20788779 | 1.374955449 | 1.092036811 | 43.57142857 | | 55.3236203 |
| hsa-miR-520b | [hsa-miR-520b](http://genome-www4.stanford.edu/cgi-bin/SMD/source/sourceResult?choice=Gene&option=Name&criteria=hsa-miR-520b) | | 0.874639982 | 0.613419243 | 0.701339128 | 1.391165332 | 43.57142857 | | 55.55297075 |
| hsa-miR-195 | [hsa-miR-195](http://genome-www4.stanford.edu/cgi-bin/SMD/source/sourceResult?choice=Gene&option=Name&criteria=hsa-miR-195) | | 0.805572242 | 0.409246891 | 0.5080201 | 1.272014769 | 43.57142857 | | 59.95085763 |
| hsa-miR-18a | [hsa-miR-18a](http://genome-www4.stanford.edu/cgi-bin/SMD/source/sourceResult?choice=Gene&option=Name&criteria=hsa-miR-18a) | | 0.797609204 | 0.570825586 | 0.71567076 | 1.244461835 | 43.57142857 | | 60.48687166 |
| hsa-miR-188 | [hsa-miR-188](http://genome-www4.stanford.edu/cgi-bin/SMD/source/sourceResult?choice=Gene&option=Name&criteria=hsa-miR-188) | | 0.768298495 | 0.474077216 | 0.617048217 | 1.228157028 | 43.57142857 | | 62.49698368 |
| hsa-miR-20a | [hsa-miR-20a](http://genome-www4.stanford.edu/cgi-bin/SMD/source/sourceResult?choice=Gene&option=Name&criteria=hsa-miR-20a) | | 0.725709662 | 0.465872037 | 0.641953748 | 1.231550517 | 43.57142857 | | 65.49040028 |
| hsa-miR-192 | [hsa-miR-192](http://genome-www4.stanford.edu/cgi-bin/SMD/source/sourceResult?choice=Gene&option=Name&criteria=hsa-miR-192) | | 0.716872491 | 0.502104889 | 0.700410317 | 1.400284014 | 43.57142857 | | 66.11716373 |
| hsa-miR-532 | [hsa-miR-532](http://genome-www4.stanford.edu/cgi-bin/SMD/source/sourceResult?choice=Gene&option=Name&criteria=hsa-miR-532) | | 0.704578517 | 0.440360804 | 0.624998909 | 1.443280499 | 43.57142857 | | 66.98970701 |
| hsa-miR-19a | [hsa-miR-19a](http://genome-www4.stanford.edu/cgi-bin/SMD/source/sourceResult?choice=Gene&option=Name&criteria=hsa-miR-19a) | | 0.704006344 | 0.508270205 | 0.721968217 | 1.171428772 | 43.57142857 | | 67.03030781 |
| hsa-miR-17-3p | [hsa-miR-17-3p](http://genome-www4.stanford.edu/cgi-bin/SMD/source/sourceResult?choice=Gene&option=Name&criteria=hsa-miR-17-3p) | | 0.676840023 | 0.34964562 | 0.51658532 | 1.181278846 | 43.57142857 | | 68.95235754 |
| hsa-miR-155 | [hsa-miR-155](http://genome-www4.stanford.edu/cgi-bin/SMD/source/sourceResult?choice=Gene&option=Name&criteria=hsa-miR-155) | | 0.624390169 | 0.321106355 | 0.514271958 | 1.281450258 | 43.57142857 | | 72.60108358 |
| hsa-miR-29b | [hsa-miR-29b](http://genome-www4.stanford.edu/cgi-bin/SMD/source/sourceResult?choice=Gene&option=Name&criteria=hsa-miR-29b) | | 0.605318127 | 0.39208622 | 0.647735798 | 1.287328044 | 43.57142857 | | 73.90577705 |
| hsa-miR-148a | [hsa-miR-148a](http://genome-www4.stanford.edu/cgi-bin/SMD/source/sourceResult?choice=Gene&option=Name&criteria=hsa-miR-148a) | | 0.579173753 | 0.333409866 | 0.575664668 | 1.310106954 | 43.57142857 | | 75.6731497 |
| hsa-miR-19b | [hsa-miR-19b](http://genome-www4.stanford.edu/cgi-bin/SMD/source/sourceResult?choice=Gene&option=Name&criteria=hsa-miR-19b) | | 0.573028074 | 0.332178443 | 0.579689649 | 1.092705431 | 43.57142857 | | 76.08382347 |
| hsa-miR-142-5p | [hsa-miR-142-5p](http://genome-www4.stanford.edu/cgi-bin/SMD/source/sourceResult?choice=Gene&option=Name&criteria=hsa-miR-142-5p) | | 0.56787896 | 0.28728149 | 0.505885074 | 1.194063144 | 43.57142857 | | 76.42617989 |
| hsa-miR-429 | [hsa-miR-429](http://genome-www4.stanford.edu/cgi-bin/SMD/source/sourceResult?choice=Gene&option=Name&criteria=hsa-miR-429) | | 0.556940717 | 0.342635967 | 0.615210842 | 1.120237381 | 43.57142857 | | 77.14778579 |
| PREDICTED_MIR207 | [PREDICTED_MIR207](http://genome-www4.stanford.edu/cgi-bin/SMD/source/sourceResult?choice=Gene&option=Name&criteria=PREDICTED_MIR207) | | 0.547636609 | 0.342414175 | 0.625258007 | 1.240859116 | 43.57142857 | | 77.75523375 |
| hsa-miR-92 | [hsa-miR-92](http://genome-www4.stanford.edu/cgi-bin/SMD/source/sourceResult?choice=Gene&option=Name&criteria=hsa-miR-92) | | 0.546479345 | 0.303806589 | 0.555934257 | 1.182848497 | 43.57142857 | | 77.83036619 |
| hsa-miR-601 | [hsa-miR-601](http://genome-www4.stanford.edu/cgi-bin/SMD/source/sourceResult?choice=Gene&option=Name&criteria=hsa-miR-601) | | 0.543783782 | 0.495733777 | 0.911637665 | 1.18869932 | 43.57142857 | | 78.00499811 |
| PREDICTED_MIR112 | [PREDICTED_MIR112](http://genome-www4.stanford.edu/cgi-bin/SMD/source/sourceResult?choice=Gene&option=Name&criteria=PREDICTED_MIR112) | | 0.537518704 | 0.315589336 | 0.587122521 | 1.174902261 | 43.57142857 | | 78.40884411 |
| hsa-miR-30e-5p | [hsa-miR-30e-5p](http://genome-www4.stanford.edu/cgi-bin/SMD/source/sourceResult?choice=Gene&option=Name&criteria=hsa-miR-30e-5p) | | 0.537309482 | 0.467488334 | 0.870054129 | 1.118660121 | 43.57142857 | | 78.42228064 |
| hsa-miR-196b | [hsa-miR-196b](http://genome-www4.stanford.edu/cgi-bin/SMD/source/sourceResult?choice=Gene&option=Name&criteria=hsa-miR-196b) | | 0.527863958 | 0.591444232 | 1.120448219 | 0.774138585 | 43.57142857 | | 79.02544787 |
| hsa-let-7c | [hsa-let-7c](http://genome-www4.stanford.edu/cgi-bin/SMD/source/sourceResult?choice=Gene&option=Name&criteria=hsa-let-7c) | | 0.525402529 | 0.35994993 | 0.685093638 | 1.048423232 | 43.57142857 | | 79.18150295 |
| NewU6-R | [NewU6-R](http://genome-www4.stanford.edu/cgi-bin/SMD/source/sourceResult?choice=Gene&option=Name&criteria=NewU6-R) | | 0.501921763 | 0.406695495 | 0.810276671 | 1.372000707 | 43.57142857 | | 80.64556372 |
| hsa-miR-29a | [hsa-miR-29a](http://genome-www4.stanford.edu/cgi-bin/SMD/source/sourceResult?choice=Gene&option=Name&criteria=hsa-miR-29a) | | 0.431195234 | 0.269427738 | 0.624839322 | 1.220282351 | 43.57142857 | | 84.77477205 |
| hsa-miR-181d | [hsa-miR-181d](http://genome-www4.stanford.edu/cgi-bin/SMD/source/sourceResult?choice=Gene&option=Name&criteria=hsa-miR-181d) | | 0.415303106 | 0.252538691 | 0.608082837 | 1.041189598 | 43.57142857 | | 85.64838119 |
| hsa-miR-150 | [hsa-miR-150](http://genome-www4.stanford.edu/cgi-bin/SMD/source/sourceResult?choice=Gene&option=Name&criteria=hsa-miR-150) | | 0.41200937 | 0.236719549 | 0.574548946 | 1.296973535 | 43.57142857 | | 85.82711129 |
| hsa-miR-29c | [hsa-miR-29c](http://genome-www4.stanford.edu/cgi-bin/SMD/source/sourceResult?choice=Gene&option=Name&criteria=hsa-miR-29c) | | 0.409760915 | 0.350906528 | 0.856368957 | 1.026417434 | 43.57142857 | | 85.94866143 |
| hsa-miR-486 | [hsa-miR-486](http://genome-www4.stanford.edu/cgi-bin/SMD/source/sourceResult?choice=Gene&option=Name&criteria=hsa-miR-486) | | 0.343735809 | 0.190913261 | 0.55540696 | 1.168725202 | 43.57142857 | | 89.35532128 |
| hsa-miR-26a | [hsa-miR-26a](http://genome-www4.stanford.edu/cgi-bin/SMD/source/sourceResult?choice=Gene&option=Name&criteria=hsa-miR-26a) | | 0.324769485 | 0.193442123 | 0.595628999 | 1.092208356 | 43.57142857 | | 90.28007519 |
| hsa-miR-564 | [hsa-miR-564](http://genome-www4.stanford.edu/cgi-bin/SMD/source/sourceResult?choice=Gene&option=Name&criteria=hsa-miR-564) | | 0.316854161 | 0.234950345 | 0.741509418 | 1.108362476 | 43.57142857 | | 90.65964899 |
| hsa-miR-186 | [hsa-miR-186](http://genome-www4.stanford.edu/cgi-bin/SMD/source/sourceResult?choice=Gene&option=Name&criteria=hsa-miR-186) | | 0.290523042 | 0.309976514 | 1.066960168 | 0.802831579 | 43.57142857 | | 91.8928164 |
| hsa-miR-23b | [hsa-miR-23b](http://genome-www4.stanford.edu/cgi-bin/SMD/source/sourceResult?choice=Gene&option=Name&criteria=hsa-miR-23b) | | 0.288356912 | 0.183143694 | 0.635128502 | 1.269091758 | 43.57142857 | | 91.99181922 |
| hsa-miR-141 | [hsa-miR-141](http://genome-www4.stanford.edu/cgi-bin/SMD/source/sourceResult?choice=Gene&option=Name&criteria=hsa-miR-141) | | 0.283685209 | 0.237290646 | 0.836457591 | 0.925640427 | 43.57142857 | | 92.20386329 |
| PREDICTED_MIR165 | [PREDICTED_MIR165](http://genome-www4.stanford.edu/cgi-bin/SMD/source/sourceResult?choice=Gene&option=Name&criteria=PREDICTED_MIR165) | | 0.268519194 | 0.161381742 | 0.601006354 | 0.991811268 | 43.57142857 | | 92.87692678 |
| hsa-miR-30b | [hsa-miR-30b](http://genome-www4.stanford.edu/cgi-bin/SMD/source/sourceResult?choice=Gene&option=Name&criteria=hsa-miR-30b) | | 0.252911663 | 0.111584562 | 0.441199747 | 1.101227062 | 43.57142857 | | 93.54108357 |
| PREDICTED_MIR166 | [PREDICTED_MIR166](http://genome-www4.stanford.edu/cgi-bin/SMD/source/sourceResult?choice=Gene&option=Name&criteria=PREDICTED_MIR166) | | 0.221204695 | 0.142289328 | 0.643247326 | 0.96105049 | 43.57142857 | | 94.7784834 |
| hsa-miR-338 | [hsa-miR-338](http://genome-www4.stanford.edu/cgi-bin/SMD/source/sourceResult?choice=Gene&option=Name&criteria=hsa-miR-338) | | 0.203723948 | 0.181630835 | 0.891553676 | 1.496518517 | 43.57142857 | | 95.38304596 |
| hsa-miR-182 | [hsa-miR-182](http://genome-www4.stanford.edu/cgi-bin/SMD/source/sourceResult?choice=Gene&option=Name&criteria=hsa-miR-182) | | 0.203396315 | 0.138166509 | 0.679297015 | 1.195189932 | 43.57142857 | | 95.39378444 |
| PREDICTED_MIR189 | [PREDICTED_MIR189](http://genome-www4.stanford.edu/cgi-bin/SMD/source/sourceResult?choice=Gene&option=Name&criteria=PREDICTED_MIR189) | | 0.157804439 | 0.107226112 | 0.6794873 | 1.060672428 | 43.57142857 | | 96.66346588 |
| hsa-miR-565 | [hsa-miR-565](http://genome-www4.stanford.edu/cgi-bin/SMD/source/sourceResult?choice=Gene&option=Name&criteria=hsa-miR-565) | | 0.156901036 | 0.105385517 | 0.671668714 | 1.053978844 | 43.57142857 | | 96.6839678 |
| hsa-miR-361 | [hsa-miR-361](http://genome-www4.stanford.edu/cgi-bin/SMD/source/sourceResult?choice=Gene&option=Name&criteria=hsa-miR-361) | | 0.130165229 | 0.061673379 | 0.473808399 | 1.030970204 | 43.57142857 | | 97.20465554 |
| hsa-miR-134 | [hsa-miR-134](http://genome-www4.stanford.edu/cgi-bin/SMD/source/sourceResult?choice=Gene&option=Name&criteria=hsa-miR-134) | | 0.123488807 | 0.067626908 | 0.547635932 | 1.030266649 | 43.57142857 | | 97.30807757 |
| hsa-miR-181b | [hsa-miR-181b](http://genome-www4.stanford.edu/cgi-bin/SMD/source/sourceResult?choice=Gene&option=Name&criteria=hsa-miR-181b) | | 0.109106625 | 0.062086802 | 0.569047037 | 1.090204389 | 43.57142857 | | 97.4934128 |
| hsa-miR-27a | [hsa-miR-27a](http://genome-www4.stanford.edu/cgi-bin/SMD/source/sourceResult?choice=Gene&option=Name&criteria=hsa-miR-27a) | | 0.045610752 | 0.023107463 | 0.506623158 | 1.034760426 | 43.57142857 | | 97.6623273 |
| hsa-miR-194 | [hsa-miR-194](http://genome-www4.stanford.edu/cgi-bin/SMD/source/sourceResult?choice=Gene&option=Name&criteria=hsa-miR-194) | | 0.03239124 | 0.02380543 | 0.734934207 | 0.99480588 | 43.57142857 | | 97.5565135 |
| hsa-miR-7 | [hsa-miR-7](http://genome-www4.stanford.edu/cgi-bin/SMD/source/sourceResult?choice=Gene&option=Name&criteria=hsa-miR-7) | | 0.01640022 | 0.016583391 | 1.011168814 | 0.90928756 | 43.57142857 | | 97.36032324 |
| **Negative genes** | | | | | | | | | |
| **Gene ID** | | **Gene Name** | **Score(d)** | **Numerator(r)** | **Denominator(s+s0)** | **Fold Change** | | **q-value(%)** | **localfdr(%)** |
| hsa-miR-181a | | [hsa-miR-181a](http://genome-www4.stanford.edu/cgi-bin/SMD/source/sourceResult?choice=Gene&option=Name&criteria=hsa-miR-181a) | -1.873312252 | -1.267054972 | 0.676371475 | 0.388572124 | | 40.66666667 | 100 |
| hsa-miR-152 | | [hsa-miR-152](http://genome-www4.stanford.edu/cgi-bin/SMD/source/sourceResult?choice=Gene&option=Name&criteria=hsa-miR-152) | -1.570311348 | -0.933891773 | 0.594717586 | 0.513125984 | | 42.890625 | 83.62804171 |
| hsa-miR-320 | | [hsa-miR-320](http://genome-www4.stanford.edu/cgi-bin/SMD/source/sourceResult?choice=Gene&option=Name&criteria=hsa-miR-320) | -1.259595919 | -0.766690968 | 0.608680099 | 0.557011284 | | 48.15789474 | 61.10513308 |
| hsa-miR-452 | | [hsa-miR-452](http://genome-www4.stanford.edu/cgi-bin/SMD/source/sourceResult?choice=Gene&option=Name&criteria=hsa-miR-452) | -1.243599755 | -0.774974086 | 0.623170021 | 0.667190547 | | 48.15789474 | 62.4396268 |
| hsa-miR-99b | | [hsa-miR-99b](http://genome-www4.stanford.edu/cgi-bin/SMD/source/sourceResult?choice=Gene&option=Name&criteria=hsa-miR-99b) | -1.207580861 | -0.741407011 | 0.613960551 | 0.550075079 | | 48.15789474 | 65.39471357 |
| hsa-miR-185 | | [hsa-miR-185](http://genome-www4.stanford.edu/cgi-bin/SMD/source/sourceResult?choice=Gene&option=Name&criteria=hsa-miR-185) | -1.183958038 | -0.524766817 | 0.443230926 | 0.709595421 | | 48.15789474 | 67.22215835 |
| hsa-miR-22 | | [hsa-miR-22](http://genome-www4.stanford.edu/cgi-bin/SMD/source/sourceResult?choice=Gene&option=Name&criteria=hsa-miR-22) | -1.13354028 | -0.655554471 | 0.578324814 | 0.626369831 | | 48.15789474 | 70.60617809 |
| hsa-miR-151 | | [hsa-miR-151](http://genome-www4.stanford.edu/cgi-bin/SMD/source/sourceResult?choice=Gene&option=Name&criteria=hsa-miR-151) | -1.108685522 | -0.548562329 | 0.494786229 | 0.667545963 | | 48.15789474 | 71.94581924 |
| hsa-miR-93 | | [hsa-miR-93](http://genome-www4.stanford.edu/cgi-bin/SMD/source/sourceResult?choice=Gene&option=Name&criteria=hsa-miR-93) | -1.008676886 | -0.634306613 | 0.628850152 | 0.634348986 | | 48.15789474 | 74.97765703 |
| hsa-miR-221 | | [hsa-miR-221](http://genome-www4.stanford.edu/cgi-bin/SMD/source/sourceResult?choice=Gene&option=Name&criteria=hsa-miR-221) | -0.964231751 | -0.404674948 | 0.419686396 | 0.741286373 | | 48.15789474 | 75.26517826 |
| hsa-miR-146b | | [hsa-miR-146b](http://genome-www4.stanford.edu/cgi-bin/SMD/source/sourceResult?choice=Gene&option=Name&criteria=hsa-miR-146b) | -0.910269894 | -0.448887035 | 0.493136198 | 0.774749225 | | 48.15789474 | 74.98992841 |
| hsa-miR-30a-5p | | [hsa-miR-30a-5p](http://genome-www4.stanford.edu/cgi-bin/SMD/source/sourceResult?choice=Gene&option=Name&criteria=hsa-miR-30a-5p) | -0.880950577 | -0.372688206 | 0.423052343 | 0.773575048 | | 48.15789474 | 74.67526458 |
| hsa-miR-100 | | [hsa-miR-100](http://genome-www4.stanford.edu/cgi-bin/SMD/source/sourceResult?choice=Gene&option=Name&criteria=hsa-miR-100) | -0.829537235 | -0.655124502 | 0.789746951 | 0.648398263 | | 48.15789474 | 74.03178478 |
| hsa-miR-106b | | [hsa-miR-106b](http://genome-www4.stanford.edu/cgi-bin/SMD/source/sourceResult?choice=Gene&option=Name&criteria=hsa-miR-106b) | -0.827858733 | -0.48668671 | 0.587886182 | 0.716753549 | | 48.15789474 | 74.01098793 |
| hsa-miR-497 | | [hsa-miR-497](http://genome-www4.stanford.edu/cgi-bin/SMD/source/sourceResult?choice=Gene&option=Name&criteria=hsa-miR-497) | -0.77512648 | -0.444452564 | 0.573393601 | 0.68612588 | | 49.95689655 | 73.42153458 |
| hsa-miR-375 | | [hsa-miR-375](http://genome-www4.stanford.edu/cgi-bin/SMD/source/sourceResult?choice=Gene&option=Name&criteria=hsa-miR-375) | -0.773994366 | -0.644903708 | 0.83321499 | 0.620254058 | | 49.95689655 | 73.41069829 |
| hsa-miR-594 | | [hsa-miR-594](http://genome-www4.stanford.edu/cgi-bin/SMD/source/sourceResult?choice=Gene&option=Name&criteria=hsa-miR-594) | -0.766026907 | -0.589396031 | 0.76941949 | 0.596847026 | | 49.95689655 | 73.33736988 |
| hsa-miR-143 | | [hsa-miR-143](http://genome-www4.stanford.edu/cgi-bin/SMD/source/sourceResult?choice=Gene&option=Name&criteria=hsa-miR-143) | -0.743869555 | -0.482967099 | 0.649263162 | 0.692992499 | | 49.95689655 | 73.16434125 |
| hsa-miR-494 | | [hsa-miR-494](http://genome-www4.stanford.edu/cgi-bin/SMD/source/sourceResult?choice=Gene&option=Name&criteria=hsa-miR-494) | -0.715197238 | -0.543282724 | 0.759626429 | 0.611953389 | | 49.95689655 | 73.02371991 |
| hsa-miR-27b | | [hsa-miR-27b](http://genome-www4.stanford.edu/cgi-bin/SMD/source/sourceResult?choice=Gene&option=Name&criteria=hsa-miR-27b) | -0.714651903 | -0.327878374 | 0.458794516 | 0.825240373 | | 49.95689655 | 73.02208711 |
| hsa-miR-125b | | [hsa-miR-125b](http://genome-www4.stanford.edu/cgi-bin/SMD/source/sourceResult?choice=Gene&option=Name&criteria=hsa-miR-125b) | -0.666242772 | -0.488848818 | 0.733739769 | 0.705772644 | | 49.95689655 | 73.03786048 |
| hsa-miR-25 | | [hsa-miR-25](http://genome-www4.stanford.edu/cgi-bin/SMD/source/sourceResult?choice=Gene&option=Name&criteria=hsa-miR-25) | -0.643930278 | -0.396734942 | 0.616114749 | 0.775413635 | | 49.95689655 | 73.15340153 |
| hsa-miR-145 | | [hsa-miR-145](http://genome-www4.stanford.edu/cgi-bin/SMD/source/sourceResult?choice=Gene&option=Name&criteria=hsa-miR-145) | -0.615505749 | -0.374855548 | 0.609020384 | 0.741310539 | | 49.95689655 | 73.401186 |
| hsa-miR-214 | | [hsa-miR-214](http://genome-www4.stanford.edu/cgi-bin/SMD/source/sourceResult?choice=Gene&option=Name&criteria=hsa-miR-214) | -0.60442425 | -0.33949594 | 0.561684844 | 0.800517234 | | 49.95689655 | 73.52862687 |
| hsa-miR-222 | | [hsa-miR-222](http://genome-www4.stanford.edu/cgi-bin/SMD/source/sourceResult?choice=Gene&option=Name&criteria=hsa-miR-222) | -0.592788056 | -0.253168082 | 0.427080269 | 0.817883158 | | 49.95689655 | 73.68112952 |
| hsa-miR-342 | | [hsa-miR-342](http://genome-www4.stanford.edu/cgi-bin/SMD/source/sourceResult?choice=Gene&option=Name&criteria=hsa-miR-342) | -0.581133944 | -0.350107827 | 0.602456337 | 0.741364537 | | 49.95689655 | 73.8530523 |
| hsa-miR-99a | | [hsa-miR-99a](http://genome-www4.stanford.edu/cgi-bin/SMD/source/sourceResult?choice=Gene&option=Name&criteria=hsa-miR-99a) | -0.575115484 | -0.428660969 | 0.745347641 | 0.805191876 | | 49.95689655 | 73.94935601 |
| hsa-miR-10a | | [hsa-miR-10a](http://genome-www4.stanford.edu/cgi-bin/SMD/source/sourceResult?choice=Gene&option=Name&criteria=hsa-miR-10a) | -0.571905084 | -0.388140378 | 0.678679713 | 0.782919768 | | 49.95689655 | 74.00282144 |
| hsa-miR-362 | | [hsa-miR-362](http://genome-www4.stanford.edu/cgi-bin/SMD/source/sourceResult?choice=Gene&option=Name&criteria=hsa-miR-362) | -0.522153206 | -0.315854144 | 0.604907028 | 0.74813141 | | 49.95689655 | 75.0182944 |
| hsa-miR-425-5p | | [hsa-miR-425-5p](http://genome-www4.stanford.edu/cgi-bin/SMD/source/sourceResult?choice=Gene&option=Name&criteria=hsa-miR-425-5p) | -0.517241365 | -0.488872136 | 0.945152822 | 0.673019805 | | 49.95689655 | 75.13795828 |
| hsa-miR-557 | | [hsa-miR-557](http://genome-www4.stanford.edu/cgi-bin/SMD/source/sourceResult?choice=Gene&option=Name&criteria=hsa-miR-557) | -0.507790892 | -0.443990808 | 0.874357565 | 0.765472681 | | 49.95689655 | 75.37840521 |
| hsa-miR-24 | | [hsa-miR-24](http://genome-www4.stanford.edu/cgi-bin/SMD/source/sourceResult?choice=Gene&option=Name&criteria=hsa-miR-24) | -0.507359611 | -0.268020329 | 0.528265009 | 0.82496682 | | 49.95689655 | 75.38970191 |
| PREDICTED_MIR191 | | [PREDICTED_MIR191](http://genome-www4.stanford.edu/cgi-bin/SMD/source/sourceResult?choice=Gene&option=Name&criteria=PREDICTED_MIR191) | -0.507202814 | -0.282720033 | 0.557410221 | 0.809970725 | | 49.95689655 | 75.39381594 |
| hsa-miR-200c | | [hsa-miR-200c](http://genome-www4.stanford.edu/cgi-bin/SMD/source/sourceResult?choice=Gene&option=Name&criteria=hsa-miR-200c) | -0.500091518 | -0.414967519 | 0.829783159 | 0.649534945 | | 49.95689655 | 75.58430892 |
| hsa-miR-210 | | [hsa-miR-210](http://genome-www4.stanford.edu/cgi-bin/SMD/source/sourceResult?choice=Gene&option=Name&criteria=hsa-miR-210) | -0.488152376 | -0.270437352 | 0.554001917 | 0.834482407 | | 49.95689655 | 75.92105085 |
| hsa-miR-370 | | [hsa-miR-370](http://genome-www4.stanford.edu/cgi-bin/SMD/source/sourceResult?choice=Gene&option=Name&criteria=hsa-miR-370) | -0.479628107 | -0.343840251 | 0.716889286 | 0.829476522 | | 49.95689655 | 76.17416246 |
| hsa-miR-130b | | [hsa-miR-130b](http://genome-www4.stanford.edu/cgi-bin/SMD/source/sourceResult?choice=Gene&option=Name&criteria=hsa-miR-130b) | -0.452665773 | -0.228393761 | 0.504552751 | 0.803207528 | | 49.95689655 | 77.04157009 |
| hsa-miR-10b | | [hsa-miR-10b](http://genome-www4.stanford.edu/cgi-bin/SMD/source/sourceResult?choice=Gene&option=Name&criteria=hsa-miR-10b) | -0.446630981 | -0.357051863 | 0.799433712 | 0.655701689 | | 49.95689655 | 77.24911665 |
| hsa-miR-21 | | [hsa-miR-21](http://genome-www4.stanford.edu/cgi-bin/SMD/source/sourceResult?choice=Gene&option=Name&criteria=hsa-miR-21) | -0.442923415 | -0.352257382 | 0.795300882 | 0.863508917 | | 49.95689655 | 77.37897587 |
| hsa-miR-200b | | [hsa-miR-200b](http://genome-www4.stanford.edu/cgi-bin/SMD/source/sourceResult?choice=Gene&option=Name&criteria=hsa-miR-200b) | -0.432263407 | -0.343449391 | 0.794537278 | 0.809071098 | | 49.95689655 | 77.7621226 |
| hsa-miR-125a | | [hsa-miR-125a](http://genome-www4.stanford.edu/cgi-bin/SMD/source/sourceResult?choice=Gene&option=Name&criteria=hsa-miR-125a) | -0.42687199 | -0.261821472 | 0.613348916 | 0.717219272 | | 49.95689655 | 77.96133652 |
| hsa-miR-610 | | [hsa-miR-610](http://genome-www4.stanford.edu/cgi-bin/SMD/source/sourceResult?choice=Gene&option=Name&criteria=hsa-miR-610) | -0.387347701 | -0.345879868 | 0.892944162 | 0.60438712 | | 49.95689655 | 79.52887845 |
| hsa-miR-484 | | [hsa-miR-484](http://genome-www4.stanford.edu/cgi-bin/SMD/source/sourceResult?choice=Gene&option=Name&criteria=hsa-miR-484) | -0.363669236 | -0.241966575 | 0.665347935 | 0.890278956 | | 49.95689655 | 80.5537847 |
| hsa-miR-638 | | [hsa-miR-638](http://genome-www4.stanford.edu/cgi-bin/SMD/source/sourceResult?choice=Gene&option=Name&criteria=hsa-miR-638) | -0.357219305 | -0.316887461 | 0.887095004 | 0.66589399 | | 49.95689655 | 80.84345735 |
| hsa-let-7i | | [hsa-let-7i](http://genome-www4.stanford.edu/cgi-bin/SMD/source/sourceResult?choice=Gene&option=Name&criteria=hsa-let-7i) | -0.346292457 | -0.166654012 | 0.481252214 | 0.910227375 | | 49.95689655 | 81.34352043 |
| hsa-miR-432 | | [hsa-miR-432](http://genome-www4.stanford.edu/cgi-bin/SMD/source/sourceResult?choice=Gene&option=Name&criteria=hsa-miR-432) | -0.307508333 | -0.154599028 | 0.502747443 | 0.865004344 | | 49.95689655 | 83.19684352 |
| hsa-miR-199a | | [hsa-miR-199a](http://genome-www4.stanford.edu/cgi-bin/SMD/source/sourceResult?choice=Gene&option=Name&criteria=hsa-miR-199a) | -0.292180749 | -0.159608133 | 0.546265056 | 0.939550781 | | 49.95689655 | 83.95814892 |
| hsa-miR-422a | | [hsa-miR-422a](http://genome-www4.stanford.edu/cgi-bin/SMD/source/sourceResult?choice=Gene&option=Name&criteria=hsa-miR-422a) | -0.225911816 | -0.147111358 | 0.651189302 | 1.007535647 | | 49.95689655 | 87.40546739 |
| PREDICTED_MIR206 | | [PREDICTED_MIR206](http://genome-www4.stanford.edu/cgi-bin/SMD/source/sourceResult?choice=Gene&option=Name&criteria=PREDICTED_MIR206) | -0.22563183 | -0.136170462 | 0.603507325 | 0.95809107 | | 49.95689655 | 87.42042946 |
| hsa-let-7b | | [hsa-let-7b](http://genome-www4.stanford.edu/cgi-bin/SMD/source/sourceResult?choice=Gene&option=Name&criteria=hsa-let-7b) | -0.222878411 | -0.153674575 | 0.689499602 | 0.791586378 | | 49.95689655 | 87.56765888 |
| hsa-miR-191 | | [hsa-miR-191](http://genome-www4.stanford.edu/cgi-bin/SMD/source/sourceResult?choice=Gene&option=Name&criteria=hsa-miR-191) | -0.208976095 | -0.148122978 | 0.708803454 | 0.767251144 | | 49.95689655 | 88.31288609 |
| hsa-miR-146a | | [hsa-miR-146a](http://genome-www4.stanford.edu/cgi-bin/SMD/source/sourceResult?choice=Gene&option=Name&criteria=hsa-miR-146a) | -0.204161382 | -0.138159655 | 0.676717867 | 1.042356319 | | 49.95689655 | 88.57137056 |
| hsa-miR-422b | | [hsa-miR-422b](http://genome-www4.stanford.edu/cgi-bin/SMD/source/sourceResult?choice=Gene&option=Name&criteria=hsa-miR-422b) | -0.184866655 | -0.115401409 | 0.624241342 | 0.99876748 | | 49.95689655 | 89.6062889 |
| hsa-miR-200a | | [hsa-miR-200a](http://genome-www4.stanford.edu/cgi-bin/SMD/source/sourceResult?choice=Gene&option=Name&criteria=hsa-miR-200a) | -0.145826985 | -0.103542242 | 0.710034856 | 0.966285463 | | 49.95689655 | 91.65508695 |
| hsa-miR-663 | | [hsa-miR-663](http://genome-www4.stanford.edu/cgi-bin/SMD/source/sourceResult?choice=Gene&option=Name&criteria=hsa-miR-663) | -0.134970939 | -0.120320348 | 0.891453736 | 0.849491579 | | 49.95689655 | 92.19941834 |
| hsa-miR-296 | | [hsa-miR-296](http://genome-www4.stanford.edu/cgi-bin/SMD/source/sourceResult?choice=Gene&option=Name&criteria=hsa-miR-296) | -0.112185142 | -0.1397173 | 1.245417148 | 0.909641364 | | 49.95689655 | 93.28908069 |
| hsa-miR-106a | | [hsa-miR-106a](http://genome-www4.stanford.edu/cgi-bin/SMD/source/sourceResult?choice=Gene&option=Name&criteria=hsa-miR-106a) | -0.073384185 | -0.043972909 | 0.59921506 | 0.878269184 | | 49.95689655 | 94.93676587 |
| hsa-miR-23a | | [hsa-miR-23a](http://genome-www4.stanford.edu/cgi-bin/SMD/source/sourceResult?choice=Gene&option=Name&criteria=hsa-miR-23a) | -0.050444585 | -0.032515763 | 0.644583807 | 1.044752423 | | 49.95689655 | 95.75831562 |
| hsa-miR-17-5p | | [hsa-miR-17-5p](http://genome-www4.stanford.edu/cgi-bin/SMD/source/sourceResult?choice=Gene&option=Name&criteria=hsa-miR-17-5p) | -0.031869678 | -0.018852584 | 0.591552399 | 0.889338767 | | 49.95689655 | 96.32627465 |
| hsa-miR-30d | | [hsa-miR-30d](http://genome-www4.stanford.edu/cgi-bin/SMD/source/sourceResult?choice=Gene&option=Name&criteria=hsa-miR-30d) | -0.027432991 | -0.012239323 | 0.44615345 | 0.965724263 | | 49.95689655 | 96.4482594 |
| hsa-miR-34a | | [hsa-miR-34a](http://genome-www4.stanford.edu/cgi-bin/SMD/source/sourceResult?choice=Gene&option=Name&criteria=hsa-miR-34a) | -0.014988017 | -0.008074278 | 0.538715567 | 0.932155093 | | 49.95689655 | 96.76175506 |

**Table S2. The** **miRBase accession number and the mature sequence of the microRNA studied**

| Accession number | ID | Sequence |
| --- | --- | --- |
| MIMAT0000256 | hsa-miR-181a-5p | AACAUUCAACGCUGUCGGUGAGU |
| MIMAT0000257 | hsa-miR-181b-5p | AACAUUCAUUGCUGUCGGUGGGU |
| MIMAT0000258 | hsa-miR-181c-5p | AACAUUCAACCUGUCGGUGAGU |
| MIMAT0002821 | hsa-miR-181d-5p | AACAUUCAUUGUUGUCGGUGGGU |

**Table S3. Clinical characteristics of colorectal cancer patients of Testing Cohort.**

| **Case** | **CRC without liver metastasis** | | | | | | | | | |
| --- | --- | --- | --- | --- | --- | --- | --- | --- | --- | --- |
|  | **1** | | | **2** | **3** | | **4** | | **5** | |
| **Gender** | | **Male** | | **Male** | **Male** | | **Female** | | **Female** | |
| **Age** | | **55** | | **56** | **56** | | **50** | | **54** | |
| **Differentiation** | | **Well** | | **Moderate** | **Well** | | **Well** | | **Moderate** | |
| **Histological type** | | **Adenocarcinoma** | | **Adenocarcinoma** | **Adenocarcinoma** | | **Adenocarcinoma** | | **Adenocarcinoma** | |
| **Lymph node metastasis** | | **Negative** | | **Negative** | **Negative** | | **Negative** | | **Negative** | |
| **liver metastasis** | | **Negative** | | **Negative** | **Negative** | | **Negative** | | **Negative** | |
| **TNM stage** | | **I** | | **П** | **I** | | **П** | | **I** | |
| **Case** | | **CRC with liver metastasis** | | | | | | | | |
|  |  | **6** | **7** | | | **8** | | **9** | | **10** |
| **Gender** | | **Male** | **Male** | | | **Male** | | **Female** | | **Female** |
| **Age** | | **52** | **54** | | | **56** | | **51** | | **53** |
| **Differentiation** | | **Poor** | **Moderate** | | | **Poor** | | **Poor** | | **Moderate** |
| **Histological type** | | **Adenocarcinoma** | **Adenocarcinoma** | | | **Adenocarcinoma** | | **Adenocarcinoma** | | **Adenocarcinoma** |
| **Lymph node metastasis** | | **Positive** | **Positive** | | | **Positive** | | **Positive** | | **Positive** |
| **liver metastasis** | | **Positive** | **Positive** | | | **Positive** | | **Positive** | | **Positive** |
| **TNM stage** | | **IV** | **IV** | | | **IV** | | **IV** | | **IV** |

**Table S4. Clinical characteristics of colorectal cancer patients of Validation Cohort 1 and 2.**

| **Clinical features** | **Cohort 1-Number** | | **Cohort 2-Number** | **P-value** |
| --- | --- | --- | --- | --- |
| **Gender** | |  |  | **0.8354** |
| **Male** | | **77** | **161** |  |
| **Female** | | **60** | **133** |  |
| **Age** | |  |  | **0.81** |
| **Mean± SD** | | **59.24 ± 1.087** | **59.55 ± 0.7194** |  |
| **Venous invasion** | |  |  | **0.8312** |
| **Positive** | | **50** | **111** |  |
| **Negative** | | **87** | **183** |  |
| **T stage** | |  |  | **0.9747** |
| **T1** | | **1** | **2** |  |
| **T2** | | **12** | **27** |  |
| **T3** | | **102** | **213** |  |
| **T4** | | **22** | **52** |  |
| **N stage** | |  |  |  |
| **N0** | | **55** | **111** | **0.8007** |
| **N1** | | **40** | **95** |  |
| **N2** | | **42** | **88** |  |
| **M stage** | |  |  |  |
| **M0** | | **60** | **97** | **0.1268** |
| **M1(synchronous liver metastasis)** | | **63** | **160** |  |
| **M1(metachronous liver metastasis)** | | **14** | **37** |  |
| **TNM stage** | |  |  | **0.4067** |
| **I** | | **12** | **22** |  |
| **П** | | **33** | **58** |  |
| **III** | | **29** | **53** |  |
| **IV** | | **63** | **161** |  |
| **Differentiation** | |  |  | **0.9637** |
| **Mucinous adenocarcinoma** | | **7** | **13** |  |
| **Poor** | | **25** | **57** |  |
| **Moderate** | | **76** | **158** |  |
| **Well** | | **29** | **66** |  |
| **Histological type** | |  |  | **0.8069** |
| **Adenocarcinoma** | | **130** | **281** |  |
| **Mucinous adenocarcinoma** | | **7** | **13** |  |
| **Lymph node metastasis** | |  |  | **0.7494** |
| **Positive** | | **83** | **184** |  |
| **Negative** | | **54** | **110** |  |

P value of less than 0.05 was considered to indicate statistical significance. P values were calculated with the use of Fisher’s exact test, except for age, which was calculated with the unpaired t-test; T stage, N stage, M stage, TNM, pathological type, and differentiation, which were calculated with the chi-square test;

**Table S5 Primer sequences**

| *Q-PCR analysis of WIF-1* | | | |
| --- | --- | --- | --- |
| GAPDH forward primer | | 5′- TGCACCACCAACTGCTTAGC -3′ | |
| GAPDH reverse primer | | 5′- GGCATGGACTGTGGTCATGAG -3′ | |
| WIF-1 forward primer | | 5′- GAAGGTTGGCATGGAAGA-3′ | |
| WIF-1 reverse primer | | 5′- TGTAATTGGATTCAGGTGGA -3′ | |
| pMIR-3’-UTR *recombinant plasmid construct* | | | |
| WIF-1-WT forward primer | 5′- CCGCTCGAGACTCCGACATCTGAAACGTTTTAAGTT 3' | | |
| WIF-1-WT reverse primer | 5′- GAATGCGGCCGCCAGAAAACTAAAGCAGCACCTTTATTTTAT 3' | | |
| WIF-1-Mut forward primer | 5'- CATGTGTAGTAAGATCAAATAATGTTCATTACA 3' | | |
| WIF-1-Mut reverse primer | 5' -TTTGATCTTACTACACATGAAAGGTTAACA 3' | | |
| The sequence and hybridization temperature of probes for *In Situ Hybridization Analysis* | | | |
| miRNA | | LNA probe sequence | Tm( ºC) |
| miR-181a | | 5’Dig-cTcaCcgAcaGcgTtgTatCtt-3’ | 76 |
| U6 | | 5’Dig-caCgaAtttgCgtGtcAtcCtt-3’ | 74 |
| Scrambled DNA | | 5’Dig-ttcaCaaTgcGttAtcCgaTgt-3’ | 75 |
